# Seasonal Dynamics of the Gut Microbiota of Ayu (*Plecoglossus altivelis*) Revealed by a Cross-Sectional Seasonal Survey in the Dajing Stream, Zhejiang Province, China

**DOI:** 10.3390/biology15080605

**Published:** 2026-04-11

**Authors:** Yuqian Wu, Heng Xu, Haichuan Li, Hufeng Chen, Libing Zhang, Shahid Ali, Jinyuan Che, Baolong Bao

**Affiliations:** 1 Shanghai Universities Key Laboratory of Marine Animal Taxonomy and Evolution, Shanghai Ocean University, Shanghai 200092, China; m230100065@st.shou.edu.cn (Y.W.); 15380686273@163.com (H.L.); 2 Yueqing Municipal Agricultural and Rural Development Center, Yueqing, Wenzhou 325600, China; hengxu2026@sina.com (H.X.); chf0824@sina.com (H.C.); 13968728091@163.com (L.Z.); 3 Department of Organismal Biology and Anatomy, The University of Chicago, Chicago, IL 60637, USA; shahidali@uchicago.edu

**Keywords:** diet composition, gut content microbiota, gut tissue-associated microbiota, *Plecoglossus altivelis*, seasonality, water microbiota

## Abstract

Ayu (*Plecoglossus altivelis*) is an East Asian amphidromous river fish, yet seasonal microbiota dynamics remain unclear. We investigated ayu in the Dajing Stream (Zhejiang Province, China) by synchronously sampling water microbiota (H), gut content microbiota (N), and gut tissue-associated microbiota (C) across four seasons. Each season, four fish were collected, and an overlapping pooling strategy (abc/abd/bcd) generated three composite replicates for C and N (*n* = 3 composites/season); water was collected as three field replicates (*n* = 3/season), yielding 36 samples (12 per niche). Using 16S rRNA amplicon sequencing and COI barcoding of stomach contents, we observed the clearest seasonal differentiation in H and seasonal variation in N consistent with diet shifts, whereas C was comparatively stable. COI signals indicated a diet dominated by aquatic insects in spring/summer, which shifted toward smaller prey (e.g., rotifers) in winter. Together, these results highlight strong niche partitioning and season-linked shifts in water and gut content communities relative to the more stable tissue-associated microbiota. These findings should be interpreted as exploratory and require validation in larger individual-level studies.

## Simple Summary

This study examined how bacterial communities in river water, ayu gut contents, and ayu gut tissue changed across four seasons in the Dajing Stream, Zhejiang Province, China. By combining 16S rRNA sequencing with DNA barcoding of stomach contents, we found that seasonal change was strongest in the water community, intermediate in gut contents, and weakest in the tissue-associated gut community. Dietary DNA signals suggested that ayu fed mainly on aquatic insects in spring and summer but shifted toward smaller prey, such as rotifers, in winter. Because the study used limited seasonal sampling and overlapping pooled gut samples, the results should be viewed as exploratory. Even so, they provide a useful baseline for future studies on ayu ecology, gut microbial stability, and stream health.

## 1. Introduction

The gastrointestinal microbiota of fish plays important roles in nutrient acquisition, energy metabolism, immune regulation, and intestinal barrier maintenance and is closely linked to the host’s capacity to cope with environmental stress [1,2,3]. Under natural conditions, gut microbial communities are shaped by both host physiology and environmental exposure, and their spatiotemporal dynamics can provide valuable insights into fish ecological adaptation and health. In aquaculture and disease management, manipulating microbial communities has been proposed as a potential strategy to reduce disease risk [4]. From a niche perspective, mucosa-associated communities are more likely to establish relatively stable colonization under host-driven selection, whereas communities in gut contents are more directly exposed to environmental inputs and can shift rapidly with short-term changes in feeding, diet composition, and the surrounding water microbiota [5,6]. Therefore, analyzing mucosa-associated microbiota, gut content microbiota, and water microbiota in parallel helps to disentangle the relative contributions of host selection and external inputs on the same temporal scale.

River-basin ecosystems show pronounced seasonal variation in water temperature, discharge, and primary productivity, which can drive seasonal shifts in fish habitat use, feeding intensity, and physiological state. Seasonal effects on gut microbiota have been reported in multiple fish species. For instance, the native gut microbiota of *Salvelinus namaycush* differs across seasons [7], and seasonal habitat migration in *Salvelinus alpinus* is accompanied by changes in gut community structure [8]. The gut microbiota of *Tinca tinca* is shaped by both habitat and season [9], while work on farmed *Gadus morhua* suggests that temporal community turnover may exceed the effects of short-term dietary supplementation [10]. In addition, winter phenology and energy-storage strategies are considered key processes in freshwater fish ecology [11,12]; by altering feeding behavior and resource availability, they may further influence gut microbial turnover and functional potential [13]. Moreover, after transfer from seawater to freshwater, gut microbiota and inflammation-related indicators can shift in parallel over short time scales [14]. Collectively, these studies indicate that across fish systems showing marked seasonal ecological transitions, seasonal habitat switching, diet reorganization, and energetic transitions tend to generate faster turnover in environmentally exposed or luminal communities, whereas host-associated communities can retain a more stable core under mucosal selection.

Ayu, *Plecoglossus altivelis*, is a common amphidromous fish in coastal rivers of East Asia and supports important commercial and recreational fisheries in southeastern China [15]. Diverse taxa, including purple non-sulfur photosynthetic bacteria, have been isolated from the ayu digestive tract, indicating a diverse gut microbial assemblage [16]. Amphidromous fishes have declined in many regions, and river fragmentation, especially dam-related fragmentation, is a major driver [17]. The life history of ayu has been documented in multiple rivers [18]. After summer growth, adults migrate downstream from autumn to early winter in association with declining water temperature and hydrological conditions and spawn in downstream gravel spawning grounds [19,20]. Hatched larvae drift to estuaries and disperse into coastal waters for overwintering [21,22], and juveniles subsequently enter and use freshwater reaches for feeding, growth, and maturation [23,24]. Environmental DNA (eDNA) approaches have been used to track ayu spatiotemporal activity in spawning grounds and river reaches [25,26]. In the Yandang Mountain region of Zhejiang Province, ayu were historically abundant in streams such as the Dajing Stream, but declines have been observed in some reaches in association with channel engineering, weirs, and changes in water quality and temperature [15]. Hydraulic structures such as drops and weirs can negatively affect the upstream migration of ayu [27]. In this context, characterizing ayu responses to seasonal environmental fluctuations from a microbial-ecological perspective may provide quantifiable indicators of ecological processes. For amphidromous ayu, seasonal upstream feeding, autumn reproductive transition, and winter downstream redistribution should repeatedly alter exposure to waterborne microbes and prey, while still allowing a host-filtered mucosal microbiota to remain comparatively stable.

Seasonal dynamics of gut microbiota in wild ayu remain poorly documented, particularly studies that jointly compare gut tissue-associated microbiota, gut content microbiota, and environmental water microbiota at the same spatiotemporal scale. Here, we conducted a cross-sectional seasonal survey of wild ayu from the Dajing Stream (Zhejiang Province, China) across spring, summer, autumn, and winter, profiling gut tissue-associated microbiota (C), gut content microbiota (N), and water microbiota (H) using 16S rRNA amplicon sequencing. Stomach contents were additionally analyzed using cytochrome c oxidase subunit I (COI) barcoding to assess seasonal diet shifts and their potential links to microbial variation.

We hypothesized that seasonal turnover would be strongest in water microbiota, intermediate in gut content microbiota, and weakest in gut tissue-associated microbiota, reflecting progressively stronger host filtering from H to N to C.

Our primary outcome was seasonal beta diversity differentiation among these three niches, whereas COI profiles were used as complementary evidence for seasonal shifts in food inputs.

## 2. Materials and Methods

### 2.1. Sampling Design and Sample Types

Ayu (*Plecoglossus altivelis*) were sampled across four seasons in the Dajing Stream, Zhejiang Province, China: summer (August 2024), autumn (October 2024), winter (December 2024), and spring (April 2025). To accommodate seasonal shifts in spatial distribution while maintaining comparability, sampling was conducted within nearby reaches of the same river system (linear distance ~ 4.6–5.4 km): upper–middle reaches in summer and autumn (121.087306° E, 28.404917° N), lower reaches in winter (121.142472° E, 28.407944° N), and an adjacent reach in spring (121.112682° E, 28.439686° N). Four individuals were collected per season, with similarly sized fish preferentially selected. Because autumn and winter catches remained limited despite repeated field effort, four fish per season were used to maintain a balanced seasonal design across all four seasons. This limited seasonal availability is broadly consistent with the strong spatiotemporal redistribution of amphidromous ayu and the practical constraints of field sampling in a stream influenced by habitat fragmentation and engineered river structures. Seasonal comparisons therefore represent cross-sectional snapshots rather than repeated sampling of the same cohort. Total length (TL) and standard length (SL) were measured using an ichthyometer (precision 1.0 mm), and body weight (BW) was measured using a balance (precision 0.1 g; 0.01 g for small individuals). The selected TL/BW ranges were spring 8.1–8.2 cm/3.7–4.1 g; summer 11.5–12.5 cm/14.5–16.5 g; autumn 12.0–13.2 cm/15.5–18.0 g; and winter 12.2–13.4 cm/16.0–19.0 g. No formal exclusion criteria were predefined, and all captured fish with sufficient material were included in the analyses. Sex and maturity status were not incorporated as analytical factors because reproductive tissues were not consistently identifiable during dissection and no histological examination was performed.

Environmental water samples were collected synchronously at each reach (2 L per replicate) and filtered through 0.22 μm membranes to capture water microbiota (H; *n* = 3 replicates per season). Given the shallow water depth, water was not stratified by depth, and sediments were not sampled. Basic physicochemical variables were measured during the seasonal surveys and are provided for contextual description only; they were not included as covariates in the statistical analyses. These variables included TN (0.344–1.577 mg/L), NO_3_-N (0.061–0.770 mg/L), NH_3_-N (0.001–0.132 mg/L), TP (0.003–0.040 mg/L), conductivity (39.7–59.3 μS/cm), DO (9.27–10.05 mg/L), salinity (0.02–0.03 psu), turbidity (0.66–8.87 FNU), pH (7.87–8.83), water temperature (11.45–28.85 °C), and transparency (38.0–56.8 cm).

### 2.2. Separation of Gut Tissue-Associated Microbiota and Gut Contents

After aseptic dissection, gut contents were separated from gut tissue. Gut contents were collected as gut content microbiota (N), and gut tissue was rinsed with sterile saline and used as gut tissue-associated microbiota (C). Stomach contents were collected for diet analysis (D). Because seasonal fish availability remained limited under standardized field sampling, only four fish could be obtained per season while maintaining a balanced design across all four seasons. Under these constraints, and given the need to generate matched material for gut tissue-associated microbiota (C), gut content microbiota (N), and diet (D) analyses, three partially overlapping pooled composites were generated per season using the combinations abc, abd, and bcd (*n* = 3). The same pooling combinations were applied to C, N, and D to enable season-scale comparisons between diet signals and microbiota patterns. These pooled composites were used to generate season-representative profiles for exploratory comparison and were not intended to represent fully independent individual-level replicates. Water microbiota (H) were processed as independent field replicates (*n* = 3 per season) without pooling. An individual-level seasonal analysis based on only four fish per season would have provided limited statistical power and insufficient matched material for parallel C, N, and D assays; the overlapping pooling design was therefore used to obtain exploratory season-level composite profiles while reducing the influence of any single fish.

### 2.3. Sequencing and Statistical Analysis

#### 2.3.1. DNA Extraction and PCR Amplification

Genomic DNA was extracted using the MagPure Soil DNA LQ Kit (Magen, Guangzhou, China) following the manufacturer’s instructions. DNA concentration and purity were assessed using a NanoDrop 2000 (Thermo Fisher Scientific, Waltham, MA, USA) spectrophotometer and agarose gel electrophoresis, and DNA was stored at −20 °C. The V3–V4 region of the 16S rRNA gene was amplified using primers 343F (5′-TACGGRAGGCAGCAG-3′) and 798R (5′-AGGGTATCTAATCCT-3′). The V3–V4 region was selected because it provides broad bacterial taxonomic coverage and has been widely used for profiling freshwater and gut-associated microbiota. PCR products were checked by agarose gel electrophoresis, purified with AMPure XP beads (Beckman Coulter, Brea, CA, USA), and subjected to a second PCR for library construction. Purified libraries were quantified using a Qubit fluorometer (Thermo Fisher Scientific, Waltham, MA, USA) and sequenced on an Illumina NovaSeq 6000 platform (Illumina Inc., San Diego, CA, USA).

#### 2.3.2. Bioinformatic and Statistical Analysis

Library construction, sequencing, and primary analyses were performed by Shanghai OE Biotech Co., Ltd. (Shanghai, China). Raw reads were generated in FASTQ format. After demultiplexing, primers/adapters were removed [28]. Quality filtering, denoising, merging, and chimera removal were performed using QIIME 2 (v2020.11) [29] with DADA2 [30], generating amplicon sequence variants (ASVs) and feature tables. Taxonomic assignment was conducted using q2-feature-classifier in QIIME 2 (v2020.11) against the SILVA database (version 138) [31]. Sequencing depth prior to rarefaction was summarized from the non-rarefied feature table (minimum 38,342 reads); for diversity analyses, the ASV table was rarefied to an even depth of 30,673 reads per sample. No additional ASV prevalence filtering was applied beyond DADA2 denoising and chimera removal. Alpha diversity indices (e.g., Shannon, Simpson, and Chao1) and beta diversity metrics (Bray–Curtis dissimilarity and UniFrac distances) were calculated in QIIME 2; principal coordinate analysis (PCoA) was used for ordination. Group comparisons of alpha diversity were evaluated using Kruskal–Wallis tests. Overall Kruskal–Wallis *p*-values, including PD_whole_tree, are provided in Supplementary Table S3a, and exact pairwise post hoc *p*-values are provided in Supplementary Table S3b. Seasonal and niche effects on beta diversity were evaluated using PERMANOVA (adonis, 999 permutations), and dispersion homogeneity was assessed using PERMDISP, with permutation schemes detailed in Supplementary Table S5. Differentially abundant taxa were identified using LEfSe [32] with α = 0.05 and an LDA score threshold of 2.0. Genus-level biomarkers and LDA effect sizes are provided in Supplementary Table S1.

### 2.4. COI Barcoding of Stomach Contents (Diet Identification)

A fragment of the mitochondrial cytochrome c oxidase subunit I (COI) gene was amplified from stomach-content DNA using primers mlCOIintF (5′-GGWACWGGWTGAACWGTWTAYCCYCC-3′) and jgHCO2198 (5′-TAIACYTCIGGRTGICCRAARAAYCA-3′). PCR products were verified by agarose gel electrophoresis, purified, and sequenced on the MGI DNBSEQ-G99 platform (MGI Tech Co., Ltd., Shenzhen, China; paired-end 300 bp).

Raw reads were demultiplexed by barcode and quality-filtered using a 10 bp sliding window; reads were truncated when the average quality score dropped below 20, and reads shorter than 50 bp were discarded. Raw COI sequencing depth per sample ranged from 100,054 to 129,758 read pairs (median 117,115). Paired-end reads were merged with a minimum overlap of 10 bp and a maximum mismatch ratio of ≤0.2, and chimeras were removed to generate feature sequences and abundance tables. Taxonomic assignment was performed by BLASTn (NCBI BLAST+ 2.15.0) against curated reference databases including MitoFish and NCBI nt, using an e-value threshold of 1 × 10^−10^ and best-hit assignment with identity/coverage filtering. Before downstream analyses, host-assigned sequences (annotated to *Plecoglossus* or Osmeriformes) were removed, and the remaining non-host reads were re-normalized for seasonal comparisons of diet composition. Host-assigned sequences refer to COI reads annotated to *Plecoglossus* or broader Osmeriformes matches, representing host carryover in stomach-content extracts rather than prey signals.

## 3. Results

### 3.1. Bacterial 16S rRNA Gene Diversity and Community Composition

**Figure 1:**
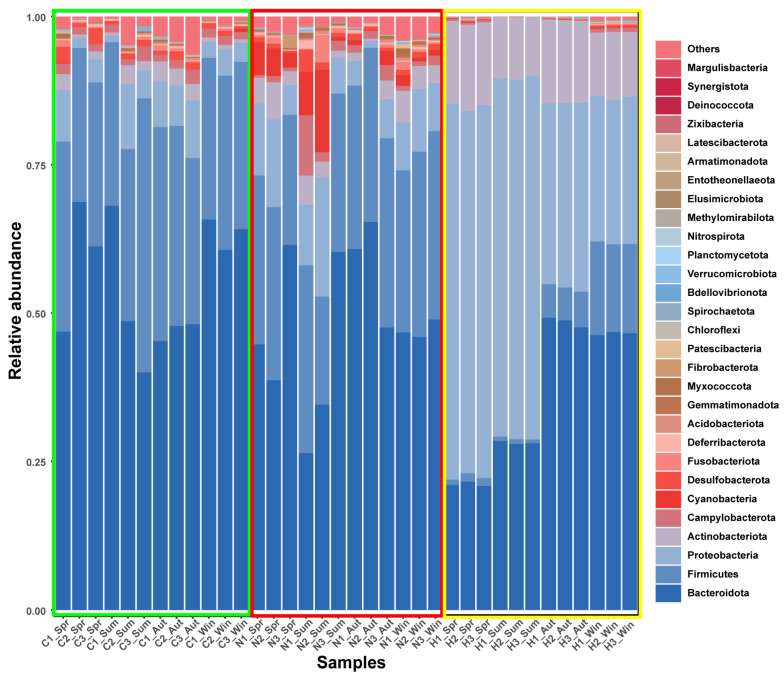
Relative abundances of bacterial communities at the phylum level across four seasons in three ecological niches. Note: Spring, Summer, Autumn and Winter are denoted as Spr, Sum, Aut and Win, respectively; C, N and H represent gut tissue-associated microbiota, gut content microbiota and water microbiota, respectively. The green boxes indicate C, red boxes indicate N, and yellow boxes indicate H; numbers represent sample labels.

A total of 1,879,876 quality-filtered non-chimeric reads were obtained from 16S rRNA gene sequencing, yielding 18,965 amplicon sequence variants (ASVs) across 36 samples. Per-sample sequencing depth and rarefied depth are summarized in Supplementary Table S2. In the non-rarefied ASV table, per-sample sequencing depth ranged from 38,342 to 62,707 reads (median 52,380; IQR 48,823–56,587). For diversity analyses, the ASV table was rarefied to an even depth of 30,673 reads per sample. Rarefaction curves based on Observed ASVs (observed features) for gut tissue-associated microbiota (C), gut content microbiota (N), and water microbiota (H) are provided in Supplementary Figures S1–S3. No additional ASV prevalence filtering was applied beyond DADA2 denoising and chimera removal. The seasonal community compositions of the three ecological niches (C, N, and H) at the phylum level are shown in Figure 1.

**Figure 2:**
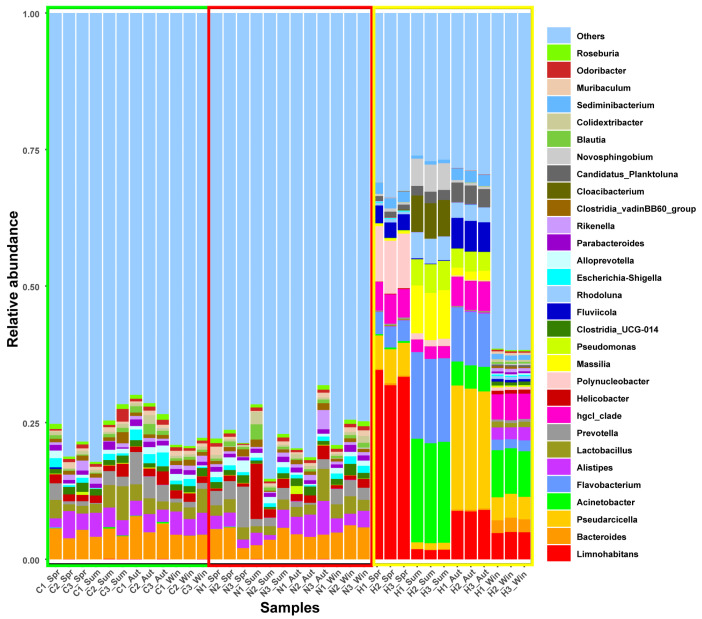
Relative abundances of bacterial communities at the genus level across four seasons in three ecological niches. Note: Spring, Summer, Autumn and Winter are denoted as Spr, Sum, Aut and Win, respectively; C, N and H represent gut tissue-associated microbiota, gut content microbiota and water microbiota, respectively. Green boxes indicate C, red boxes indicate N, and yellow boxes indicate H; numbers represent sample labels.

For water microbiota (H) at the phylum level, Proteobacteria was the dominant phylum in spring and summer, with Actinobacteriota maintaining a relatively high proportion; Bacteroidota dominated in autumn and winter, and the relative abundance of Firmicutes increased in winter (Figure 1). At the genus level, *Limnohabitans* was the dominant genus in spring, with a relatively high abundance of *Polynucleobacter*; *Acinetobacter* and *Flavobacterium* were the main dominant genera in summer; *Pseudarcicella* dominated in autumn, accompanied by a relatively high abundance of *Fluviicola*; and *Acinetobacter* and *Limnohabitans* were the major dominant genera in winter, with a relatively high abundance of *Pseudarcicella* (Figure 2).

For gut content microbiota (N), at the phylum level, Bacteroidota and Firmicutes were the dominant phyla across all four seasons. The proportion of Proteobacteria increased in summer, along with elevated relative abundances of Cyanobacteria and Campylobacterota (Figure 1). At the genus level, *Bacteroides* and *Prevotella* were the main dominant genera in spring; *Helicobacter* dominated in summer, with *Bacteroides* still accounting for a relatively high proportion; *Alistipes* was the dominant genus in autumn, and the relative abundance of *Lactobacillus* increased; *Bacteroides* was the major dominant genus in winter, with *Prevotella*, *Lactobacillus* and *Alistipes* as important constituent genera (Figure 2).

For gut tissue-associated microbiota (C) at the phylum level, the community was primarily composed of Bacteroidota and Firmicutes across all four seasons, with a relatively stable overall structure (Figure 1). At the genus level, *Bacteroides* was the core dominant genus in all seasons; the relative abundance of *Lactobacillus* increased in summer; the relative abundance of *Prevotella* rose in autumn; *Bacteroides* remained the dominant genus in winter, with *Alistipes* and *Lactobacillus* as key constituent genera (Figure 2).

### 3.2. Bacterial Alpha Diversity and Inter-Sample Comparisons

Alpha diversity indices of each group are shown in Table 1, whereas the complete set of overall Kruskal–Wallis *p*-values, including PD_whole_tree, is provided in Supplementary Table S3a, and the exact pairwise post hoc *p*-values are provided in Supplementary Table S3b. Kruskal–Wallis tests across seasons indicated significant seasonal differences only in the water microbiota (H), including observed richness (*p* = 0.04148), Chao1 (*p* = 0.04148), Shannon (*p* = 0.02162), and Simpson (*p* = 0.01556) (Table 1), as well as PD_whole_tree (*p* = 0.02488) (Supplementary Table S3a). In contrast, seasonal differences were not significant for gut content microbiota (N) or gut tissue-associated microbiota (C), although seasonal trends were observed (minimum *p* = 0.12068 for N and 0.18342 for C; Supplementary Table S3a).

### 3.3. Bacterial Beta Diversity (PCoA) and Community Differentiation

Principal coordinate analysis (PCoA) based on Bray–Curtis dissimilarities showed the clearest seasonal separation in water microbiota (H), moderate separation in gut content microbiota (N), and substantial overlap in gut tissue-associated microbiota (C) across the four seasons (Supplementary Figures S4–S6).

PERMANOVA (adonis, 999 permutations) indicated that season explained community variation in H (R^2^ = 0.98323; df = 3, 8; pseudo-F = 156.37; *p* = 0.001), N (R^2^ = 0.33314; df = 3, 8; pseudo-F = 1.3322; *p* = 0.021), and C (R^2^ = 0.33349; df = 3, 8; pseudo-F = 1.3342; *p* = 0.118) (Supplementary Table S4). Homogeneity of dispersion among seasons was additionally evaluated using PERMDISP on the original Bray–Curtis distance matrices, with permutation schemes detailed in Supplementary Table S5, showing significant dispersion differences in H (F = 26.0949; df = 3, 8; *p* = 0.010), but not in N (F = 3.8588; df = 3, 8; *p* = 0.386) or C (F = 8.5803; df = 3, 8; *p* = 0.292) (Supplementary Table S5). The H compartment exhibited the most pronounced seasonal differentiation across beta diversity analyses, indicating a robust seasonal signal in water-associated communities. At the same time, variation in within-group dispersion suggests that this pattern may reflect both seasonal separation and differences in within-season heterogeneity.

Within each season, the three ecological niches (H, N, and C) also differed significantly in community structure based on Bray–Curtis distances, with PERMANOVA R^2^ values ranging from 0.53480 to 0.65267 (Supplementary Table S4). Shared ASVs among the three niches (H ∩ N ∩ C) were quantified within each season, yielding 74 shared ASVs in spring, 36 in summer, 96 in autumn, and 197 in winter, indicating increased niche overlap during winter; these values were summarized from the ASV feature tables (Supplementary Table S6). These shared-ASV counts provide a descriptive view of seasonal niche overlap and are consistent with the stronger winter convergence pattern observed in the beta diversity analyses; the seasonal overlap plots are provided in Supplementary Figure S7 for visualization.

**Figure 3:**
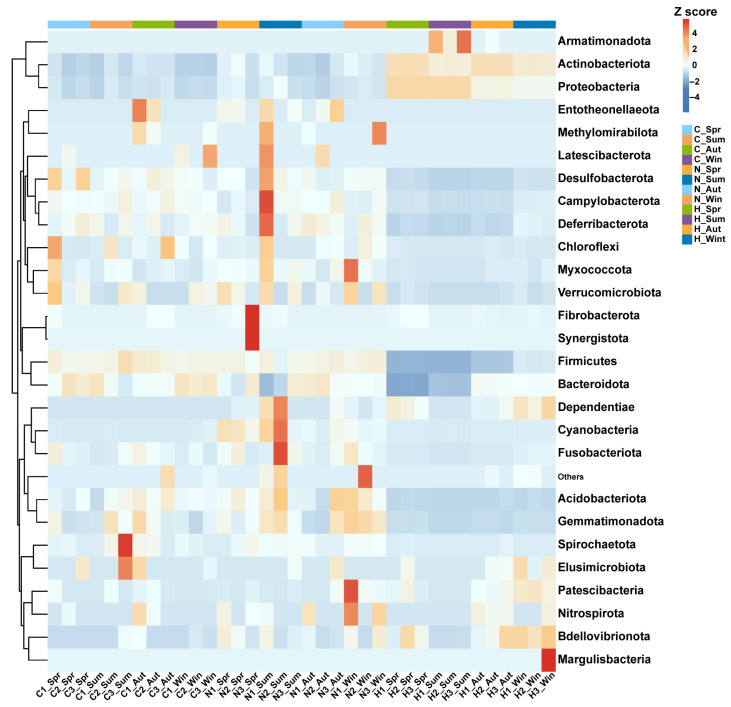
Dominant bacterial community structure at the phylum level in three niches across four seasons. Note: Spring, Summer, Autumn, and Winter are denoted as Spr, Sum, Aut, and Win, respectively. C, N, and H represent gut tissue-associated microbiota, gut content microbiota, and water microbiota, respectively. Numbers indicate sample labels.

**Figure 4:**
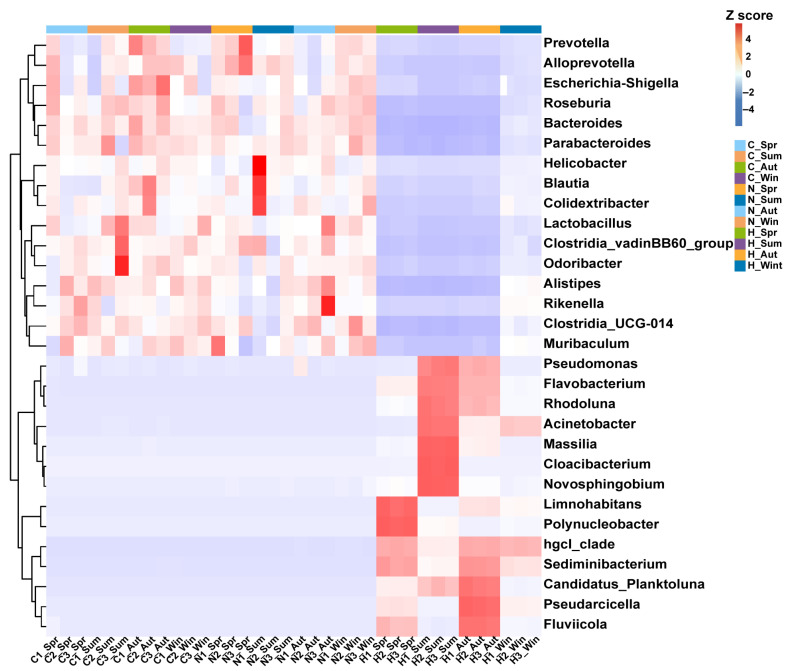
Dominant bacterial community structure at the genus level in three niches across four seasons. Note: Spring, Summer, Autumn, and Winter are denoted as Spr, Sum, Aut, and Win, respectively. C, N, and H represent gut tissue-associated microbiota, gut content microbiota, and water microbiota, respectively. Numbers indicate sample labels.

Heatmaps at the phylum and genus levels are shown in Figure 3 and Figure 4. In hierarchical clustering, H samples grouped more consistently by season, whereas N and C samples were more dispersed (Figure 3 and Figure 4).

LEfSe identified genus-level biomarker taxa, and LDA effect sizes together with *p*-values are reported in Supplementary Table S1. More biomarkers were detected in H, with *Limnohabitans* and *Polynucleobacter* enriched in spring, *Acinetobacter* and *Flavobacterium* in summer, *Pseudarcicella* and *Fluviicola* in autumn, and *Rheinheimera*, *Alistipes*, and *Bacteroides* in winter. In N, biomarkers included *Prevotella* and *Alloprevotella* in spring and *Candidatus_Arthromitus* and *Dialister* in winter. In C, biomarkers included *Clostridium_sensu_stricto_1*, *Shuttleworthia*, and *Peptoniphilus* in autumn and *Ruminiclostridium* and *Ellin6067* in winter.

### 3.4. Dietary Differences (COI)

**Figure 5:**
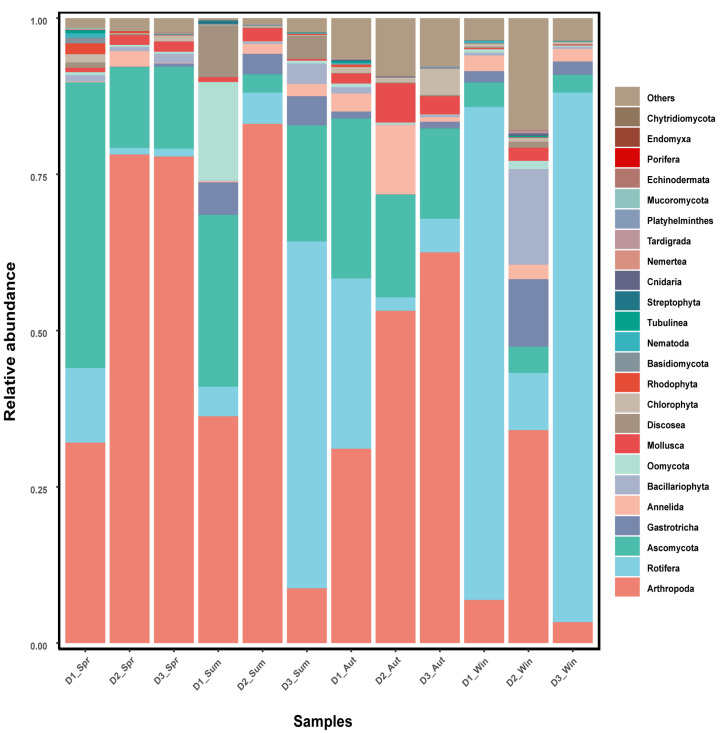
Relative abundance of dietary composition of *Plecoglossus altivelis* at the phylum level across four seasons (COI). Note: Spring, Summer, Autumn and Winter are denoted as Spr, Sum, Aut and Win, respectively. Numbers indicate sample labels.

**Figure 6:**
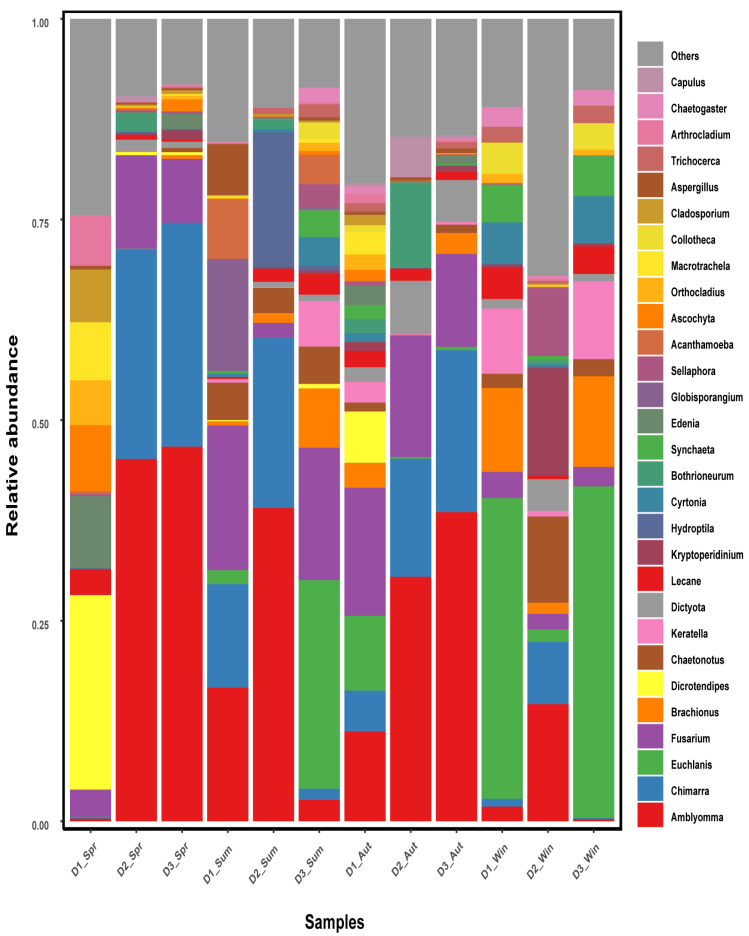
Relative abundance of dietary composition of *Plecoglossus altivelis* at the genus level across four seasons (COI). Note: Spring, Summer, Autumn and Winter are denoted as Spr, Sum, Aut and Win, respectively. Numbers indicate sample labels.

COI sequencing yielded 100,054–129,758 clean read pairs per sample (median 117,115.5). After ASV construction and chimera removal, 89,497–116,033 reads per sample (median 95,658.5) were retained in the feature table. Host-assigned reads, annotated to *Plecoglossus* or Osmeriformes, accounted for 75.63% of retained reads overall, ranging from 28.98% to 98.36% across samples, and averaged 94.52% in spring, 92.61% in summer, 63.42% in autumn, and 49.14% in winter (Supplementary Table S7). After host removal, the remaining non-host reads ranged from 1706 to 69,156 per sample (median 10,921.5), and these non-host reads were used for subsequent seasonal comparisons (Supplementary Table S7). Bray–Curtis PERMANOVA indicated significant seasonal differences in the non-host COI profiles (df = 3, 8; pseudo–F = 2.870157; R^2^ = 0.518376; *p* = 0.010), whereas PERMDISP, using the permutation scheme detailed in Supplementary Table S5, was not significant (F = 4.0873; df = 3, 8; *p* = 0.279) (Supplementary Tables S4 and S5). The resulting seasonal non–host taxonomic compositions are shown in Figure 5 and Figure 6. The lower host-read proportion in winter likely reflects a larger contribution of prey-derived and environmentally derived DNA in stomach extracts after the shift toward small planktonic prey, whereas high spring-summer host fractions are consistent with stronger host carryover relative to non-host diet signals. Because all seasonal diet comparisons were conducted after explicit host-sequence removal and re-normalization of the remaining reads, this difference in host carryover should mainly affect sensitivity for detecting low-abundance prey rather than the direction of dominant seasonal patterns.

At the phylum level, Arthropoda was the dominant group in spring, summer, and autumn, whereas Rotifera dominated in winter. At the genus level, the dominant genera differed among seasons: in spring, *Dicrotendipes*, *Amblyomma*, and *Chimarra* were predominant; in summer, *Amblyomma*, *Chimarra*, and *Fusarium*; in autumn, *Amblyomma*, *Chimarra*, and *Fusarium*; and in winter, *Euchlanis*, *Brachionus*, and *Keratella*.

## 4. Discussion

In this study, we compared the water microbiota (H), gut content microbiota (N), and gut tissue-associated microbiota (C) across spring, summer, autumn, and winter in the Dajing Stream catchment, Zhejiang Province, China, and used COI-based diet data to complement information on seasonal food sources. The three communities remained distinct within each season, and the magnitude of seasonal variation decreased from H to N to C, with the gut tissue-associated microbiota showing the highest stability. This pattern suggests that the gut content community is more readily influenced by environmental microbes and diet, whereas the mucosa-associated community is more strongly constrained by host filtering and therefore varies less across seasons [33]. From the perspective of niche mechanisms, gut tissue-associated microbiota tend to form relatively stable colonizing communities on the mucosal surface.

### 4.1. Increased External Inputs in Spring

In spring, the water microbiota (H) was dominated by Proteobacteria at the phylum level and by *Limnohabitans* and *Polynucleobacter* at the genus level. Both *Limnohabitans* and *Polynucleobacter* are common heterotrophic taxa in freshwater bacterioplankton [34,35]. Members of these lineages are associated with rapid use of algal-derived dissolved organic matter and other labile carbon pools, and they are not restricted to the free-water phase but may also occur in particle-associated or periphytic assemblages [36,37]. In freshwater systems, periphyton and related biofilms provide a plausible matrix through which phototroph–heterotroph interactions can accelerate the turnover of exuded and polymeric organic matter [37].

The gut content microbiota (N) in spring was dominated by Bacteroidota and Firmicutes at the phylum level, with *Bacteroides* and *Prevotella* as the dominant genera. COI-based diet analysis indicated that spring diets were mainly composed of arthropods, including aquatic insects such as *Dicrotendipes* and *Chimarra*. Arthropod-derived material and associated biofilms can introduce complex polysaccharides, proteins, attached particles, and exogenous microbes into the digestive tract [5]. When aquatic arthropods are ingested together with attached periphyton and biofilm-coated surfaces, these water-associated taxa, or their metabolic signals, may enter the gut lumen as transient inputs rather than stable colonizers. Fish guts contain both relatively stable mucosa-associated communities and more transient diet- and water-associated microbial inputs; accordingly, externally derived signals are more likely to appear first in the lumen-facing gut-content microbiota (N) than to establish immediately as stable members of the tissue-associated microbiota (C) [38]. Because gut-content microbiota (N) is directly exposed to ingested material and environmental microbes, whereas gut tissue-associated microbiota (C) is more strongly buffered by host filtering and mucosal selection, this pathway is expected to influence N more rapidly than C [33].

The gut tissue-associated microbiota (C) in spring was dominated by Bacteroidota and Firmicutes at the phylum level and was mainly composed of *Bacteroides*, *Alistipes*, and *Lactobacillus* at the genus level. Proteobacteria remained at a relatively low proportion, consistent with a more stable mucosa-associated community. The repeated dominance of *Bacteroides* in C across seasons is consistent with a core host-associated guild adapted to the utilization of host- and diet-derived glycans at the mucosal interface [39]. Such glycan metabolism can support fermentation-derived metabolite production, including short-chain fatty acids, and is functionally linked to maintenance of mucosal barrier integrity and immune homeostasis [40]. This contrast between a rapidly responsive lumen-facing compartment (N) and a more buffered tissue-associated compartment (C) is consistent with the spring pattern observed here.

### 4.2. Intensified Feeding in Summer

In summer, taxa such as *Acinetobacter* and *Flavobacterium* increased in the water microbiota (H). Summer warming generally intensifies microbial metabolism and organic-matter turnover in stream environments, while biofilm growth, sloughing, and particle-associated processing become more dynamic [37,41]. In running waters, warming may enhance biofilm turnover and particle-associated microbial processing, potentially favoring opportunistic heterotrophs in the water column [37,41]. *Flavobacterium*-related signals have also been reported in ayu health-monitoring studies [42], indicating that the summer enrichment observed in H may reflect a seasonal shift in the environmental microbial background encountered by fish. This pattern is therefore discussed here in the context of warm-season environmental exposure and changing external microbial conditions. Because fish-pathogenic *Flavobacterium* spp. in freshwater systems have been associated with epithelial surface disease, including skin lesions, fin erosion, and gill necrosis or inflammation, this summer enrichment may also be relevant to external mucosal health and host-condition monitoring in ayu [43]. COI results indicated that arthropods remained the main food source in summer. Meanwhile, Cyanobacteria and Campylobacterota increased in the gut content microbiota (N), which may be related to higher feeding intensity, dietary composition, and more frequent water contact during the warm season [5,6].

In the gut tissue-associated microbiota (C), *Bacteroides*, *Alistipes*, and *Lactobacillus* remained the core dominant genera, whereas Proteobacteria stayed at a low proportion, consistent with mucosal filtering of exogenous microbial signals [33]. Fermentation-related gut microbes are often associated with short-chain fatty acid production and may contribute to maintaining mucosal barrier function and intestinal homeostasis in vertebrate gut systems, including teleosts [40,44]. For ayu, summer often coincides with rapid growth under concurrent environmental stress [19]. The persistence of core taxa in the mucosal compartment may help maintain fermentative metabolism and barrier stability under high temperatures and elevated feeding levels, thereby reducing the impact of short-term external fluctuations on the host. Within this stable mucosal core, persistent dominance of *Bacteroides* is consistent with continued utilization of host- and diet-derived glycans and with sustained production of fermentation-derived metabolites that support epithelial barrier stability and immune homeostasis [39,40]. This functional role may help buffer the tissue-associated compartment from short-term environmental fluctuations even under warm-season conditions with intensified feeding and stronger external microbial inputs.

### 4.3. Autumn Transition

In autumn, the water microbiota (H) shifted from Proteobacteria dominance in spring–summer to a higher proportion of Bacteroidota, with increases in taxa such as *Pseudarcicella* and *Fluviicola*, indicating that the environmental microbial pool differed from spring–summer. COI results showed that arthropods remained the major food items in autumn, mainly represented by aquatic-insect-related taxa, while fungal or terrestrial signals were detected in some samples.

In the gut content microbiota (N), *Bacteroides* remained dominant, and taxa such as *Alistipes* increased, consistent with seasonal changes in substrate spectra and environmental exposure [11]. Some ayu begin downstream movement in autumn, and temperature and discharge may influence their spatial distribution and seasonal habitat use [19]. These patterns are discussed primarily in the context of known ayu life history and riverine environmental dynamics. River barriers can reduce habitat connectivity and constrain migration pathways, and previous work has shown that weirs may lower passage efficiency in ayu [24].

In the gut tissue-associated microbiota (C), *Bacteroides*, *Alistipes*, and *Lactobacillus* remained dominant, while *Prevotella* increased in autumn. Autumn is also a period of reproductive transition in ayu, and lower temperature together with changing discharge conditions may promote downstream redistribution [20]. During field sampling, individuals were obtained mainly from lower reaches in autumn and winter, which is broadly consistent with seasonal downstream movement reported for ayu, although movement and connectivity were not directly measured here. The Dajing Stream system also includes engineered river structures (e.g., sluice gates and weirs), which may influence flow conditions and habitat continuity and thereby alter environmental inputs relevant to microbial communities. Increases in *Prevotella* and the continued dominance of *Bacteroides* in autumn are more plausibly interpreted in relation to shifts in substrate availability, fermentation strategies, and mucosa-associated resource use [39,45]. Seasonal cooling and reproductive transition may alter both the spectrum of dietary and host-derived glycans and the competitive balance within the community. Because 16S amplicon data are compositional, the observed relative enrichment may reflect not only more favorable substrates for these taxa but also, in part, compositional rebalancing as other taxa decline [46]. The continued dominance of *Bacteroides* in autumn, likewise, suggests persistent mucosa-associated glycan use and fermentation-linked support of epithelial stability during reproductive transition [39,40]. The autumn increase in *Prevotella* is therefore broadly consistent with a host-physiology-linked shift in mucosal regulation and substrate availability during reproductive transition.

### 4.4. Winter Low-Temperature Convergence

In winter, the proportions of Bacteroidota and Firmicutes further increased in the water microbiota (H), and the number of shared ASVs with dominant gut taxa also increased. Low temperature and reduced discharge can alter particle retention and microbial transport in the stream. Under low-flow winter conditions, weaker dilution and longer water residence can favor the accumulation and recurrent resuspension of fine particulate organic matter and detached biofilm fragments near the benthos. This process may increase the availability of environment-derived microbial material in the near-bottom water column and facilitate its entry into the digestive tract during winter feeding and water contact [37,38]. COI results showed that winter diets shifted toward small planktonic prey such as rotifers, and *Bacteroides* increased in relative abundance in the gut content microbiota (N). Together, these observations suggest that the larger winter overlap is associated with low-flow-driven particle retention and recurrent resuspension of environment-derived particulate and biofilm-associated microbes that can enter the gut as transient inputs during winter.

Under low-temperature stress, the gut tissue-associated microbiota (C) remained centered on *Bacteroides*, *Alistipes*, and *Lactobacillus*, indicating maintenance of mucosal homeostasis. The continued dominance of *Bacteroides* in winter further suggests that this genus functions as a stable mucosal core member capable of sustaining glycan utilization, fermentation-derived metabolite production, and immune-supportive host–microbiota interactions even under low-temperature stress [39,40]. Taken together, the repeated dominance of *Bacteroides* across spring, summer, autumn, and winter supports its interpretation as a year-round core mucosal taxon in ayu. Across seasons, this persistence is consistent with a conserved role in host- and diet-derived glycan utilization, fermentation-linked metabolite production, and support of epithelial and intestinal homeostasis. Nevertheless, diversity in C was lower in winter than in autumn, and LEfSe detected modest shifts in a small number of responsive biomarker taxa, suggesting that the mucosal compartment is not static but adjusts within a limited range to maintain stability. Environmental conditions can also affect the risk of pathogen-associated mortality in ayu [47]. Winter cooling and low-flow conditions can change activity, feeding, and the composition of food inputs, thereby altering the substrates and microbial signals entering the gut [11]. Previous work in ayu has shown that perturbations of gut microbial communities can interact with host immune responses [48], and fecal or gut-derived omics signals have been explored as indicators of fish condition [42]. In the present study, the winter convergence between H and C, together with the modest fluctuations detected in C, suggests that winter may be an informative season for evaluating intestinal stability under changing environmental inputs. In this context, winter appears to represent a season in which external microbial inputs, mucosal stability, and host-associated core taxa can be considered together when interpreting the ecological dynamics of the ayu gut microbiota.

### 4.5. Study Limitations

Using this cross-sectional seasonal design, the present study provides an initial exploratory view of seasonal microbiota variation in ayu by jointly comparing water microbiota, gut content microbiota, and gut tissue-associated microbiota. The analyses consistently indicated stronger seasonal turnover in water microbiota, intermediate seasonal variation in gut content microbiota, and comparatively greater stability in the tissue-associated gut community, thereby providing an exploratory seasonal baseline for understanding ayu gut microbial ecology in the Dajing Stream catchment. This baseline should be interpreted in light of the present season-level sampling constraints, including limited biological replication in autumn and winter, the use of partially overlapping pooled composites for gut-associated samples, and the absence of some controls and analytical extensions. Future studies with larger individual-level sampling, time-matched environmental measurements, negative extraction, PCR controls, mock communities, blinding procedures, and analytical approaches such as compositional transformations including CLR and nested or hierarchical modeling frameworks may further refine these patterns and allow more rigorous inference on seasonal and niche-related effects.

## 5. Conclusions

This study characterized seasonal microbiota dynamics of ayu in the Dajing Stream watershed by jointly profiling water microbiota (H), gut content microbiota (N), and gut tissue-associated microbiota (C). Water microbiota showed the strongest seasonal turnover, and gut content microbiota showed an intermediate degree of turnover, whereas gut tissue-associated microbiota remained comparatively stable across seasons. These patterns, together with COI-inferred seasonal diet shifts, highlight strong niche partitioning and suggest that variation in external inputs may contribute more strongly to gut content dynamics than to tissue-associated communities. Overall, the results provide an initial microbial-ecological baseline for future work on ayu ecology and stream-associated microbial dynamics. Future larger-scale, individual-level studies will help to confirm and extend these findings. These conclusions should nevertheless be read in light of the pooled design and limited seasonal replication. Despite these limitations, from a management perspective, season-specific monitoring of water and gut content microbiota may help identify periods when environmentally driven microbial turnover is strongest and when ayu are most exposed to changing external microbial inputs. In addition, maintaining flow connectivity and habitat quality may help buffer abrupt environmental transitions during migration and overwintering, thereby supporting more stable host-associated microbial conditions.

## Figures and Tables

**Table 1:** Alpha diversity indices of the three ecological niches across four seasons (mean ± standard deviation).

Season	Ecological Niche	Observed Species	Chao1	Shannon	Simpson
Spring	H (Water microbiota)	455.03 ± 141.75	462.09 ± 143.31	6.78 ± 0.25	0.98 ± 0.00
Summer	H (Water microbiota)	324.13 ± 26.41	327.27 ± 27.79	6.57 ± 0.07	0.98 ± 0.00
Autumn	H (Water microbiota)	464.67 ± 60.98	473.92 ± 63.27	6.13 ± 0.13	0.94 ± 0.00
Winter	H (Water microbiota)	690.37 ± 61.48	698.03 ± 63.14	7.88 ± 0.08	0.99 ± 0.00
Spring	N (Gut content microbiota)	1223.77 ± 103.53	1231.08 ± 102.26	8.98 ± 0.65	0.99 ± 0.01
Summer	N (Gut content microbiota)	1019.67 ± 156.64	1023.46 ± 157.05	8.20 ± 0.16	0.99 ± 0.00
Autumn	N (Gut content microbiota)	879.60 ± 335.26	884.37 ± 339.11	8.05 ± 0.93	0.99 ± 0.01
Winter	N (Gut content microbiota)	982.63 ± 140.62	987.53 ± 141.92	9.17 ± 0.07	1.00 ± 0.00
Spring	C (Gut tissue-associated microbiota)	718.97 ± 61.77	722.17 ± 60.62	8.00 ± 0.64	0.99 ± 0.00
Summer	C (Gut tissue-associated microbiota)	816.43 ± 285.72	821.11 ± 286.86	8.37 ± 0.13	0.99 ± 0.01
Autumn	C (Gut tissue-associated microbiota)	1065.90 ± 236.15	1074.19 ± 237.17	9.14 ± 0.29	1.00 ± 0.00
Winter	C (Gut tissue-associated microbiota)	784.57 ± 175.31	788.28 ± 175.43	7.71 ± 0.44	0.99 ± 0.00

## Data Availability

The data supporting this study are available at https://doi.org/10.6084/m9.figshare.31314769 (accessed on 2 April 2026). Raw sequencing reads have been deposited in the NCBI Sequence Read Archive (SRA) under BioProject accession PRJNA1430197.

## Supplementary Materials

The following supporting information can be downloaded at: https://www.mdpi.com/article/10.3390/biology15080605/s1. Table S1. Genus-level biomarkers and LDA effect sizes identified by LEfSe; Table S2. Per-sample sequencing depth and rarefied depth for 16S rRNA data; Table S3a. Overall Kruskal–Wallis *p*-values for alpha-diversity indices; Table S3b. Pairwise post hoc *p*-values for alpha-diversity indices; Table S4. PERMANOVA results; Table S5. PERMDISP results and permutation schemes; Table S6. Seasonal shared ASV counts among water microbiota (H), gut content microbiota (N), and gut tissue-associated microbiota (C); Table S7. COI read counts before and after host-sequence removal; Figure S1. Rarefaction curves for gut tissue-associated microbiota (C); Figure S2. Rarefaction curves for gut content microbiota (N); Figure S3. Rarefaction curves for water microbiota (H); Figure S4. PCoA plot for seasonal beta-diversity patterns in H; Figure S5. PCoA plot for seasonal beta-diversity patterns in N; Figure S6. PCoA plot for seasonal beta-diversity patterns in C; Figure S7. Seasonal overlap plots showing shared and unique ASVs among H, N, and C.
