# Supplementary material for: Seasonal Dynamics of the Gut Microbiota of Ayu (Plecoglossus altivelis) Revealed by a Cross-Sectional Seasonal Survey in the Dajing Stream, Zhejiang Province, China
Source: Biology (Basel). 2026 Apr 11;15(8):605. doi: 10.3390/biology15080605 (PMC13114198; doi:10.3390/biology15080605)
Supplement: Supplementary file 1 [file biology-15-00605-s001.zip › SuppTable S1-S7/SuppTable_S1_LEfSe.pdf]

Supplementary Table S1. LEfSe-selected differential biomarker taxa with corresponding LDA effect sizes and p-values.

Group	Genus	LDA	p-value
C-Aut	Actibacter	2.424	0.0132489965279209
C-Aut	Candidatus_Stoquefichus	2.542	0.0132489965279209
C-Win	Ruminiclostridium	2.708	0.0402857078711178
C-Aut	Clostridium_sensu_stricto_1	3.045	0.0476285147084644
C-Aut	Shuttleworthia	2.749	0.0421640767150978
C-Aut	Peptoniphilus	2.713	0.040513478090991
C-Win	Ellin6067	2.522	0.0132489965279209
C-Aut	Proteus	2.688	0.0421640767150978
C-Aut	o__Acidobacteriales	2.918	0.0269915810798503
C-Aut	f__Acidobacteriaceae__Subgroup_1_	2.713	0.0132489965279209
C-Aut	o__Bacillales	3.235	0.0474500454504873
C-Aut	f__Bacillaceae	3.160	0.0422156125457654
C-Aut	f__Peptostreptococcales_Tissierellales	3.217	0.030254651790181
C-Aut	c__Longimicrobia	2.694	0.0132489965279209
C-Aut	o__Longimicrobiales	2.704	0.0132489965279209
C-Aut	f__Longimicrobiaceae	2.693	0.0132489965279209
C-Aut	f__Sutterellaceae	3.476	0.0434350841537281
N-Spr	Klenkia	3.362	0.0132489965279209
N-Spr	Chryseoglobus	3.472	0.0132489965279209
N-Spr	Alloprevotella	3.761	0.0434350841537281
N-Spr	Prevotella	4.244	0.0299805369160345
N-Spr	Prevotellaceae_UCG_003	3.909	0.0200555676960257
N-Spr	Fibrobacter	3.597	0.0325381936165011
N-Win	Christensenellaceae_R_7_group	3.051	0.0155643974585932

Group	Genus	LDA	p-value
N-Win	Candidatus_Arthromitus	3.477	0.0217064651464967
N-Win	Dialister	3.034	0.0324476431503432
N-Spr	Bosea	3.647	0.0236573493561056
N-Spr	Methylobacterium_Methylorubrum	3.431	0.0254191830349234
N-Spr	Allorhizobium_Neorhizobium_Pararhizobium_Rhizobium	3.355	0.0444198331305124
N-Spr	Rubellimicrobium	3.325	0.0132489965279209
N-Sum	o__Microtrichales	3.085	0.0329015914281892
N-Spr	f__Geodermatophilaceae	3.554	0.0412457181882606
N-Spr	o__Micrococcales	3.692	0.0299805369160345
N-Spr	f__Microbacteriaceae	3.496	0.0412384541050273
N-Spr	o__Propionibacteriales	3.538	0.0315156165549429
N-Spr	f__Nocardioidaceae	3.532	0.03184571886305
N-Spr	p__Fibrobacterota	3.587	0.0325381936165011
N-Spr	c__Fibrobacteria	3.618	0.0325381936165011
N-Spr	o__Fibrobacterales	3.600	0.0325381936165011
N-Spr	f__Fibrobacteraceae	3.626	0.0325381936165011
N-Win	o__Christensenellales	3.070	0.0155643974585932
N-Win	f__Christensenellaceae	3.077	0.0155643974585932
N-Win	o__Clostridiales	3.518	0.044448402794751
N-Win	f__Clostridiaceae	3.531	0.044448402794751
N-Win	c__Negativicutes	3.117	0.0332126275221238
N-Win	o__Veillonellales_Selenomonadales	3.097	0.0332126275221238
N-Win	f__Veillonellaceae	3.106	0.0409395350889694
N-Spr	o__Rhodobacterales	3.914	0.044448402794751
N-Spr	f__Rhodobacteraceae	3.872	0.044448402794751
H-Spr	CL500_29_marine_group	4.151	0.0155643974585932

Group	Genus	LDA	p-value
H-Aut	hgcI_clade	4.207	0.0237440664767199
H-Aut	Candidatus_Planktoluna	4.198	0.0155643974585932
H-Sum	Rhodoluna	4.302	0.0248799450280187
H-Win	Bacteroides	4.054	0.0248799450280187
H-Win	Muribaculaceae	5.147	0.0155643974585932
H-Win	Alistipes	4.058	0.0237440664767199
H-Aut	Pseudarcicella	5.025	0.0155643974585932
H-Aut	Fluviicola	4.427	0.0155643974585932
H-Sum	Flavobacterium	4.819	0.0155643974585932
H-Sum	Chryseobacterium	4.080	0.0248799450280187
H-Win	Lachnospiraceae_NK4A136_group	4.382	0.0155643974585932
H-Sum	Novosphingobium	4.370	0.0237440664767199
H-Win	Rheinheimera	4.111	0.0137809211220399
H-Spr	Polynucleobacter	4.681	0.0155643974585932
H-Sum	Acidovorax	4.331	0.0145459555717703
H-Spr	Limnohabitans	5.191	0.0155643974585932
H-Spr	Rhodoferax	4.139	0.0155643974585932
H-Sum	Massilia	4.650	0.0155643974585932
H-Sum	Acinetobacter	4.947	0.0155643974585932
H-Sum	Pseudomonas	4.406	0.0155643974585932
H-Spr	p__Actinobacteriota	4.235	0.0248799450280187
H-Spr	c__Acidimicrobiia	4.135	0.0155643974585932
H-Spr	o__Microtrichales	4.152	0.0155643974585932
H-Spr	f__Ilumatobacteraceae	4.133	0.0155643974585932
H-Aut	c__Actinobacteria	4.376	0.0155643974585932
H-Spr	o__Frankiales	4.345	0.0155643974585932

Group	Genus	LDA	p-value
H-Spr	f__Sporichthyaceae	4.374	0.0155643974585932
H-Aut	o__Micrococcales	4.508	0.0155643974585932
H-Aut	f__Microbacteriaceae	4.499	0.0155643974585932
H-Aut	p__Bacteroidota	5.139	0.0155643974585932
H-Aut	c__Bacteroidia	5.149	0.0155643974585932
H-Win	o__Bacteroidales	5.284	0.0187853557614606
H-Win	f__Bacteroidaceae	4.065	0.0248799450280187
H-Win	f__Muribaculaceae	5.156	0.0155643974585932
H-Win	f__Rikenellaceae	4.214	0.0187853557614606
H-Spr	o__Chitinophagales	4.242	0.0155643974585932
H-Spr	f__Chitinophagaceae	4.204	0.0155643974585932
H-Aut	o__Cytophagales	5.035	0.0155643974585932
H-Aut	f__Spirosomaceae	5.002	0.0155643974585932
H-Sum	o__Flavobacteriales	5.038	0.0155643974585932
H-Aut	f__Crocinitomicaceae	4.427	0.0155643974585932
H-Sum	f__Flavobacteriaceae	4.845	0.0155643974585932
H-Aut	f__NS9_marine_group	4.002	0.0153061181890628
H-Sum	f__Weeksellaceae	4.662	0.0248799450280187
H-Win	p__Firmicutes	4.858	0.0155643974585932
H-Aut	c__Bacilli	4.345	0.0187853557614606
H-Aut	o__Bacillales	4.237	0.0137809211220399
H-Aut	f__Bacillaceae	4.247	0.0137809211220399
H-Win	c__Clostridia	4.823	0.0155643974585932
H-Win	o__Lachnospirales	4.615	0.0187853557614606
H-Win	f__Lachnospiraceae	4.636	0.0187853557614606
H-Win	o__Oscillospirales	4.199	0.0155643974585932

Group	Genus	LDA	p-value
H-Spr	p__Proteobacteria	5.290	0.0187853557614606
H-Sum	c__Alphaproteobacteria	4.778	0.0248799450280187
H-Sum	o__Caulobacterales	4.295	0.0155643974585932
H-Sum	f__Caulobacteraceae	4.313	0.0155643974585932
H-Sum	o__Rhizobiales	4.099	0.0155643974585932
H-Sum	o__Sphingomonadales	4.443	0.0155643974585932
H-Sum	f__Sphingomonadaceae	4.484	0.0155643974585932
H-Spr	c__Gammaproteobacteria	5.255	0.0155643974585932
H-Win	o__Alteromonadales	4.132	0.0145459555717703
H-Win	f__Alteromonadaceae	4.119	0.0137809211220399
H-Spr	o__Burkholderiales	5.366	0.0155643974585932
H-Spr	f__Burkholderiaceae	4.681	0.0155643974585932
H-Spr	f__Comamonadaceae	5.255	0.0155643974585932
H-Sum	f__Oxalobacteraceae	4.681	0.0155643974585932
H-Sum	o__Pseudomonadales	5.067	0.0155643974585932
H-Sum	f__Moraxellaceae	4.962	0.0155643974585932
H-Sum	f__Pseudomonadaceae	4.381	0.0155643974585932
H-Spr	CL500_29_marine_group	4.154	0.0221091284018549
H-Spr	Candidatus_Planktophila	3.620	0.0221091284018549
H-Spr	Sporichthyaceae	3.946	0.0221091284018549
H-Spr	hgcI_clade	4.411	0.0347352589447385
H-Spr	Candidatus_Planktoluna	3.685	0.0221091284018549
C-Spr	Muribaculaceae	5.271	0.0389902195744872
N-Spr	Alloprevotella	3.846	0.0389902195744872
N-Spr	Prevotella	4.310	0.0389902195744872
C-Spr	Prevotellaceae_UCG_001	4.119	0.0389902195744872

Group	Genus	LDA	p-value
C-Spr	Rikenella	3.735	0.0389902195744872
N-Spr	Rikenellaceae_RC9_gut_group	3.921	0.0496478039318924
H-Spr	Sediminibacterium	3.993	0.0221091284018549
H-Spr	Pseudarcicella	4.506	0.0221091284018549
H-Spr	Fluviicola	4.154	0.0347352589447385
C-Spr	Helicobacter	3.806	0.0273237224472925
N-Spr	Fibrobacter	3.519	0.0221091284018549
C-Spr	Lachnospiraceae_NK4A136_group	4.488	0.0389902195744872
C-Spr	Lachnospiraceae_UCG_001	3.533	0.0273237224472925
N-Spr	Bosea	3.708	0.0221091284018549
H-Spr	Polynucleobacter	4.703	0.0221091284018549
H-Spr	Limnohabitans	5.219	0.0347352589447385
H-Spr	Malikia	3.634	0.0221091284018549
H-Spr	Polaromonas	3.940	0.0221091284018549
H-Spr	Rhodoferax	4.145	0.0221091284018549
H-Spr	p__Actinobacteriota	4.811	0.0389902195744872
H-Spr	o__Microtrichales	4.173	0.0265095004990672
H-Spr	f__Ilumatobacteraceae	4.135	0.0221091284018549
H-Spr	c__Actinobacteria	4.719	0.0273237224472925
H-Spr	o__Frankiales	4.603	0.0265095004990672
H-Spr	f__Sporichthyaceae	4.601	0.0265095004990672
H-Spr	o__Micrococcales	4.073	0.0389902195744872
H-Spr	f__Microbacteriaceae	4.103	0.0265095004990672
C-Spr	f__Muribaculaceae	5.291	0.0389902195744872
H-Spr	f__Spirosomaceae	4.498	0.0221091284018549
H-Spr	f__Crocinitomicaceae	4.170	0.0347352589447385

Group	Genus	LDA	p-value
C-Spr	p__Campilobacterota	3.706	0.0273237224472925
C-Spr	c__Campylobacteria	3.754	0.0273237224472925
C-Spr	o__Campylobacterales	3.716	0.0273237224472925
C-Spr	f__Helicobacteraceae	3.803	0.0273237224472925
N-Spr	o__Cyanobacteriales	4.164	0.0459381308132181
C-Spr	o__Lachnospirales	4.853	0.0389902195744872
C-Spr	f__Lachnospiraceae	4.863	0.0389902195744872
H-Spr	p__Proteobacteria	5.482	0.0389902195744872
N-Spr	f__Beijerinckiaceae	4.023	0.0265095004990672
N-Spr	f__Rhizobiaceae	3.607	0.0496478039318924
H-Spr	f__Rhizobiales_Incertae_Sedis	3.765	0.0241338965449133
H-Sum	Sporichthyaceae	3.680	0.0221091284018549
H-Sum	hgcI_clade	4.066	0.0347352589447385
H-Sum	Candidatus_Planktoluna	3.972	0.0221091284018549
H-Sum	Rhodoluna	4.345	0.0221091284018549
N-Sum	Alloprevotella	3.686	0.0265095004990672
C-Sum	Alistipes	4.259	0.0389902195744872
H-Sum	Pseudarcicella	3.777	0.0221091284018549
H-Sum	Flavobacterium	4.856	0.0347352589447385
H-Sum	Chryseobacterium	4.101	0.0347352589447385
H-Sum	Cloacibacterium	4.532	0.0221091284018549
C-Sum	Lactobacillus	4.292	0.0379415415715289
C-Sum	Clostridia_UCG_014	3.785	0.0379415415715289
C-Sum	Clostridia_vadinBB60_group	3.758	0.0459381308132181
H-Sum	Brevundimonas	3.903	0.0347352589447385
H-Sum	Caulobacter	4.094	0.0221091284018549

Group	Genus	LDA	p-value
H-Sum	Allorhizobium_Neorhizobium_Pararhizobium_Rhizobium	3.783	0.0339856065141385
H-Sum	Shinella	3.533	0.0221091284018549
H-Sum	Novosphingobium	4.406	0.0347352589447385
H-Sum	Polynucleobacter	3.767	0.0221091284018549
H-Sum	Vogesella	3.561	0.0221091284018549
H-Sum	Acidovorax	4.321	0.0347352589447385
H-Sum	Comamonas	3.806	0.0339856065141385
H-Sum	Sphaerotilus	3.502	0.0221091284018549
H-Sum	Massilia	4.635	0.0347352589447385
H-Sum	o__Frankiales	4.217	0.0459381308132181
H-Sum	f__Sporichthyaceae	4.173	0.0339856065141385
H-Sum	f__Spirosomaceae	3.859	0.0339856065141385
H-Sum	o__Flavobacteriales	5.087	0.0273237224472925
H-Sum	f__Flavobacteriaceae	4.910	0.0273237224472925
N-Sum	c__Cyanobacteriia	4.586	0.0339856065141385
N-Sum	o__Cyanobacteriales	4.107	0.0221091284018549
N-Sum	p__Desulfobacterota	4.049	0.0496478039318924
N-Sum	c__Desulfovibrionia	4.014	0.0496478039318924
N-Sum	o__Desulfovibrionales	4.026	0.0496478039318924
N-Sum	f__Desulfovibrionaceae	3.989	0.0496478039318924
N-Sum	f__Erysipelotrichaceae	3.608	0.0348632560694255
C-Sum	f__Lactobacillaceae	4.279	0.0379415415715289
C-Sum	o__Clostridia_UCG_014	3.758	0.0379415415715289
C-Sum	f__Clostridia_UCG_014	3.763	0.0379415415715289
C-Sum	o__Clostridia_vadinBB60_group	3.754	0.0459381308132181
C-Sum	f__Clostridia_vadinBB60_group	3.794	0.0459381308132181

Group	Genus	LDA	p-value
N-Sum	p__Fusobacteriota	3.907	0.0348632560694255
N-Sum	c__Fusobacteriia	3.934	0.0348632560694255
N-Sum	o__Fusobacteriales	3.950	0.0348632560694255
N-Sum	f__Fusobacteriaceae	3.898	0.0459381308132181
H-Sum	f__Caulobacteraceae	4.315	0.0339856065141385
H-Sum	f__Rhizobiaceae	4.071	0.0496478039318924
H-Sum	o__Burkholderiales	5.018	0.0389902195744872
H-Sum	f__Chromobacteriaceae	3.527	0.0221091284018549
H-Sum	f__Methylophilaceae	3.657	0.0347352589447385
H-Sum	f__Oxalobacteraceae	4.652	0.0389902195744872
H-Aut	Candidatus_Planktoluna	4.222	0.0221091284018549
H-Aut	Rhodoluna	4.127	0.0347352589447385
C-Aut	Bacteroides	4.485	0.0273237224472925
N-Aut	Muribaculaceae	5.253	0.0389902195744872
C-Aut	Prevotella	4.317	0.0273237224472925
N-Aut	Alistipes	4.355	0.0389902195744872
H-Aut	Sediminibacterium	4.019	0.0347352589447385
H-Aut	Pseudarcicella	5.051	0.0347352589447385
H-Aut	Fluviicola	4.444	0.0221091284018549
H-Aut	Flavobacterium	4.666	0.0241338965449133
N-Aut	Lachnospiraceae_NK4A136_group	4.558	0.0389902195744872
H-Aut	Acinetobacter	4.349	0.0379415415715289
H-Aut	o__Micrococcales	4.556	0.0273237224472925
H-Aut	f__Microbacteriaceae	4.575	0.0496478039318924
N-Aut	o__Bacteroidales	5.421	0.0273237224472925
C-Aut	f__Bacteroidaceae	4.487	0.0273237224472925

Group	Genus	LDA	p-value
N-Aut	f__Muribaculaceae	5.277	0.0389902195744872
C-Aut	f__Prevotellaceae	4.617	0.0273237224472925
N-Aut	f__Rikenellaceae	4.540	0.0389902195744872
H-Aut	o__Cytophagales	5.059	0.0459381308132181
H-Aut	f__Spirosomaceae	5.052	0.0347352589447385
H-Aut	o__Flavobacteriales	4.952	0.0273237224472925
H-Aut	f__Crocinitomicaceae	4.446	0.0221091284018549
H-Aut	f__Flavobacteriaceae	4.675	0.0273237224472925
H-Aut	o__Bacillales	4.240	0.0265095004990672
H-Aut	f__Bacillaceae	4.232	0.0265095004990672
C-Aut	f__Ruminococcaceae	4.120	0.0273237224472925
H-Aut	p__Proteobacteria	5.096	0.0273237224472925
H-Aut	c__Gammaproteobacteria	5.133	0.0273237224472925
C-Aut	o__Enterobacterales	4.055	0.0273237224472925
C-Aut	f__Enterobacteriaceae	4.025	0.0273237224472925
H-Aut	f__Moraxellaceae	4.363	0.0265095004990672
H-Win	hgcI_clade	4.371	0.0339856065141385
N-Win	Bacteroides	4.170	0.0273237224472925
N-Win	Prevotella	4.026	0.0273237224472925
H-Win	Pseudarcicella	4.324	0.0221091284018549
H-Win	Rheinheimera	4.125	0.0221091284018549
H-Win	Limnohabitans	4.417	0.0347352589447385
H-Win	p__Actinobacteriota	4.701	0.0273237224472925
H-Win	c__Acidimicrobiia	4.034	0.0273237224472925
H-Win	c__Actinobacteria	4.606	0.0273237224472925
H-Win	o__Frankiales	4.544	0.0241338965449133

Group	Genus	LDA	p-value
H-Win	f__Sporichthyaceae	4.543	0.0241338965449133
C-Win	o__Bacteroidales	5.076	0.0273237224472925
N-Win	f__Bacteroidaceae	4.209	0.0273237224472925
N-Win	f__Prevotellaceae	4.255	0.0389902195744872
H-Win	f__Spirosomaceae	4.326	0.0221091284018549
H-Win	o__Flavobacteriales	4.115	0.0273237224472925
H-Win	p__Proteobacteria	5.043	0.0273237224472925
N-Win	c__Alphaproteobacteria	4.118	0.0273237224472925
H-Win	c__Gammaproteobacteria	5.006	0.0273237224472925
H-Win	f__Alteromonadaceae	4.126	0.0221091284018549
H-Win	o__Burkholderiales	4.622	0.0273237224472925
H-Win	f__Comamonadaceae	4.540	0.0273237224472925
H-Win	f__Moraxellaceae	4.618	0.0273237224472925

Note: The group codes denote niche–season combinations. H, water microbiota; N, gut content microbiota; C, gut tissue-associated microbiota. Spr, spring; Sum, summer; Aut, autumn; Win, winter.
