# Supplementary material for: Seasonal Dynamics of the Gut Microbiota of Ayu (Plecoglossus altivelis) Revealed by a Cross-Sectional Seasonal Survey in the Dajing Stream, Zhejiang Province, China
Source: Biology (Basel). 2026 Apr 11;15(8):605. doi: 10.3390/biology15080605 (PMC13114198; doi:10.3390/biology15080605)
Supplement: Supplementary file 1 [file biology-15-00605-s001.zip › SuppTable S1-S7/SuppTable_S2_16S_depth.pdf]

Supplementary Table S2. Per-sample 16S sequencing summary.

Part 1. Quality-control and denoising summary.

Sample	Season	Niche	Raw reads	Filtered	Filter pass %	Denoised	Merged	Merge %	Non-chimeric	Non-chimeric %
C-c-1	Spring	Gut tissue	79987	74509	93.15	70769	61029	76.30	58938	73.68
C-c-2	Spring	Gut tissue	78490	76383	97.32	74067	57243	72.93	50256	64.03
C-c-3	Spring	Gut tissue	79766	77162	96.74	75068	59213	74.23	53656	67.27
C-d-1	Winter	Gut tissue	79669	77529	97.31	75132	57313	71.94	50670	63.60
C-d-2	Winter	Gut tissue	79002	76072	96.29	73717	57978	73.39	52101	65.95
C-d-3	Winter	Gut tissue	81115	78414	96.67	75234	57140	70.44	49643	61.20
C-q-1	Autumn	Gut tissue	81910	77116	94.15	75143	57964	70.77	53989	65.91
C-q-2	Autumn	Gut tissue	81886	78703	96.11	76073	61764	75.43	57900	70.71
C-q-3	Autumn	Gut tissue	78695	73915	93.93	71279	62118	78.94	58997	74.97
C-x-1	Summer	Gut tissue	80016	77459	96.80	75136	57433	71.78	48456	60.56
C-x-2	Summer	Gut tissue	80596	77236	95.83	75935	64994	80.64	60468	75.03
C-x-3	Summer	Gut tissue	79639	76642	96.24	74687	60614	76.11	53937	67.73
H-c-1	Spring	Water	81001	76745	94.75	69805	56751	70.06	45885	56.65
H-c-2	Spring	Water	80821	78331	96.92	73784	61024	75.51	48873	60.47
H-c-3	Spring	Water	80535	77524	96.26	72125	60886	75.60	50876	63.17
H-d-1	Winter	Water	80637	77826	96.51	73109	54300	67.34	49174	60.98
H-d-2	Winter	Water	78077	75330	96.48	70427	52343	67.04	47206	60.46
H-d-3	Winter	Water	79880	77438	96.94	72763	55859	69.93	50840	63.65
H-q-1	Autumn	Water	78286	75667	96.65	71057	57169	73.03	50119	64.02
H-q-2	Autumn	Water	80487	77489	96.28	73322	59778	74.27	52659	65.43
H-q-3	Autumn	Water	81185	78948	97.24	74739	61697	76.00	54655	67.32
H-x-1	Summer	Water	80999	78581	97.01	76375	59149	73.02	42610	52.61
H-x-2	Summer	Water	81048	78788	97.21	76863	61774	76.22	44074	54.38
H-x-3	Summer	Water	78182	76322	97.62	74305	59992	76.73	44805	57.31
N-c-1	Spring	Gut content	81657	78892	96.61	75999	61985	75.91	56173	68.79

Sample	Season	Niche	Raw reads	Filtered	Filter pass %	Denoised	Merged	Merge %	Non-chimeric	Non-chimeric %
N-c-2	Spring	Gut content	81698	78763	96.41	76219	63125	77.27	58374	71.45
N-c-3	Spring	Gut content	79416	76227	95.98	72398	50746	63.90	38342	48.28
N-d-1	Winter	Gut content	80020	76704	95.86	75124	65575	81.95	62707	78.36
N-d-2	Winter	Gut content	78115	72091	92.29	70553	58485	74.87	55445	70.98
N-d-3	Winter	Gut content	80469	77117	95.83	75026	61921	76.95	57340	71.26
N-q-1	Autumn	Gut content	80054	77181	96.41	74829	59915	74.84	53549	66.89
N-q-2	Autumn	Gut content	78706	75687	96.16	72852	55051	69.95	47546	60.41
N-q-3	Autumn	Gut content	81271	78055	96.04	76187	63267	77.85	57339	70.55
N-x-1	Summer	Gut content	79298	76430	96.38	73551	58956	74.35	57230	72.17
N-x-2	Summer	Gut content	79829	74211	92.96	69980	58292	73.02	56372	70.62
N-x-3	Summer	Gut content	80072	77409	96.67	74127	53898	67.31	48672	60.79

Note: A total of 36 samples were retained, with 18,965 ASVs after denoising. Non-rarefied sequencing depth ranged from 38,342 to 62,707 reads (median 52,380; Q1 48,822.75; Q3 56,586.5). All samples were rarefied to 30,673 reads per sample. Percentages are reported as provided by the raw QC and feature-summary outputs.

Part 2. Feature-table depth and rarefaction summary.

Sample	Observed ASVs	Pre-rarefaction depth	Rarefied depth	Retained after rarefaction %
C-c-1	658	58938	30673	52.04
C-c-2	726	50256	30673	61.03
C-c-3	780	53656	30673	57.17
C-d-1	818	50670	30673	60.53
C-d-2	945	52101	30673	58.87
C-d-3	599	49643	30673	61.79
C-q-1	1196	53989	30673	56.81
C-q-2	1217	57900	30673	52.98
C-q-3	798	58997	30673	51.99
C-x-1	497	48456	30673	63.30
C-x-2	918	60468	30673	50.73
C-x-3	1043	53937	30673	56.87
H-c-1	324	45885	30673	66.85
H-c-2	610	48873	30673	62.76
H-c-3	442	50876	30673	60.29
H-d-1	696	49174	30673	62.38
H-d-2	631	47206	30673	64.98
H-d-3	758	50840	30673	60.33
H-q-1	397	50119	30673	61.20
H-q-2	504	52659	30673	58.25
H-q-3	512	54655	30673	56.12
H-x-1	296	42610	30673	71.99
H-x-2	349	44074	30673	69.59
H-x-3	330	44805	30673	68.46
N-c-1	1318	56173	30673	54.60

Sample	Observed ASVs	Pre-rarefaction depth	Rarefied depth	Retained after rarefaction %
N-c-2	1253	58374	30673	52.55
N-c-3	1110	38342	30673	80.00
N-d-1	966	62707	30673	48.91
N-d-2	855	55445	30673	55.32
N-d-3	1136	57340	30673	53.49
N-q-1	935	53549	30673	57.28
N-q-2	523	47546	30673	64.51
N-q-3	1191	57339	30673	53.49
N-x-1	1114	57230	30673	53.60
N-x-2	842	56372	30673	54.41
N-x-3	1113	48672	30673	63.02
