# Supplementary material for: Seasonal Dynamics of the Gut Microbiota of Ayu (Plecoglossus altivelis) Revealed by a Cross-Sectional Seasonal Survey in the Dajing Stream, Zhejiang Province, China
Source: Biology (Basel). 2026 Apr 11;15(8):605. doi: 10.3390/biology15080605 (PMC13114198; doi:10.3390/biology15080605)
Supplement: Supplementary file 1 [file biology-15-00605-s001.zip › SuppTable S1-S7/SuppTable_S3a_overall_KW.pdf]

Supplementary Table S3a. Overall exact p-values for alpha-diversity comparisons (Kruskal–Wallis tests).

| Comparison | Index | Test | H statistic | Exact p-value |
|---|---|---|---|---|
| Seasonal comparison within gut tissue microbiota | observed_species | Kruskal_Wallis | 3.8205 | 0.281509144597733 |
| Seasonal comparison within gut tissue microbiota | chao1 | Kruskal_Wallis | 3.8205 | 0.281509144597733 |
| Seasonal comparison within gut tissue microbiota | shannon | Kruskal_Wallis | 4.8462 | 0.183415417945506 |
| Seasonal comparison within gut tissue microbiota | simpson | Kruskal_Wallis | 4.8462 | 0.183415417945506 |
| Seasonal comparison within gut tissue microbiota | PD_whole_tree | Kruskal_Wallis | 1.7692 | 0.62165344399561 |
| Seasonal comparison within gut content microbiota | observed_species | Kruskal_Wallis | 3.8205 | 0.281509144597733 |
| Seasonal comparison within gut content microbiota | chao1 | Kruskal_Wallis | 4.5897 | 0.204423696054008 |
| Seasonal comparison within gut content microbiota | shannon | Kruskal_Wallis | 5.8205 | 0.120676795307346 |
| Seasonal comparison within gut content microbiota | simpson | Kruskal_Wallis | 4.7949 | 0.187448782391578 |
| Seasonal comparison within gut content microbiota | PD_whole_tree | Kruskal_Wallis | 5.3590 | 0.147321084918778 |
| Seasonal comparison within water microbiota | observed_species | Kruskal_Wallis | 8.2308 | 0.0414755598461636 |
| Seasonal comparison within water microbiota | chao1 | Kruskal_Wallis | 8.2308 | 0.0414755598461636 |
| Seasonal comparison within water microbiota | shannon | Kruskal_Wallis | 9.6667 | 0.0216228376077032 |
| Seasonal comparison within water microbiota | simpson | Kruskal_Wallis | 10.3846 | 0.0155643974585932 |
| Seasonal comparison within water microbiota | PD_whole_tree | Kruskal_Wallis | 9.3590 | 0.0248799450280188 |

Note: This table reports the overall exact p-values for each alpha-diversity index within each seasonal comparison. These values are the primary overall tests used to summarize seasonal alpha-diversity differences.
