# Supplementary material for: Seasonal Dynamics of the Gut Microbiota of Ayu (Plecoglossus altivelis) Revealed by a Cross-Sectional Seasonal Survey in the Dajing Stream, Zhejiang Province, China
Source: Biology (Basel). 2026 Apr 11;15(8):605. doi: 10.3390/biology15080605 (PMC13114198; doi:10.3390/biology15080605)
Supplement: Supplementary file 1 [file biology-15-00605-s001.zip › SuppTable S1-S7/SuppTable_S3b_pairwise_p.pdf]

Supplementary Table S3b. Pairwise exact p-values from alpha-diversity post hoc files.

Comparison	Index	Group 1	Group 2	Exact p-value	Adjustment
Seasonal comparison within gut tissue microbiota	observed_species	C-Sum	C-Spr	0.62	none
Seasonal comparison within gut tissue microbiota	observed_species	C-Aut	C-Spr	0.12	none
Seasonal comparison within gut tissue microbiota	observed_species	C-Aut	C-Sum	0.31	none
Seasonal comparison within gut tissue microbiota	observed_species	C-Win	C-Spr	0.59	none
Seasonal comparison within gut tissue microbiota	observed_species	C-Win	C-Sum	0.88	none
Seasonal comparison within gut tissue microbiota	observed_species	C-Win	C-Aut	0.18	none
Seasonal comparison within gut tissue microbiota	chao1	C-Sum	C-Spr	0.61	none
Seasonal comparison within gut tissue microbiota	chao1	C-Aut	C-Spr	0.12	none
Seasonal comparison within gut tissue microbiota	chao1	C-Aut	C-Sum	0.31	none
Seasonal comparison within gut tissue microbiota	chao1	C-Win	C-Spr	0.59	none
Seasonal comparison within gut tissue microbiota	chao1	C-Win	C-Sum	0.88	none
Seasonal comparison within gut tissue microbiota	chao1	C-Win	C-Aut	0.17	none
Seasonal comparison within gut tissue microbiota	shannon	C-Sum	C-Spr	0.678	none
Seasonal comparison within gut tissue microbiota	shannon	C-Aut	C-Spr	0.073	none
Seasonal comparison within gut tissue microbiota	shannon	C-Aut	C-Sum	0.391	none
Seasonal comparison within gut tissue microbiota	shannon	C-Win	C-Spr	0.551	none
Seasonal comparison within gut tissue microbiota	shannon	C-Win	C-Sum	0.457	none
Seasonal comparison within gut tissue microbiota	shannon	C-Win	C-Aut	0.013	none
Seasonal comparison within gut tissue microbiota	simpson	C-Sum	C-Spr	0.8	none
Seasonal comparison within gut tissue microbiota	simpson	C-Aut	C-Spr	0.14	none
Seasonal comparison within gut tissue microbiota	simpson	C-Aut	C-Sum	0.4	none
Seasonal comparison within gut tissue microbiota	simpson	C-Win	C-Spr	0.58	none
Seasonal comparison within gut tissue microbiota	simpson	C-Win	C-Sum	0.55	none
Seasonal comparison within gut tissue microbiota	simpson	C-Win	C-Aut	0.02	none
Seasonal comparison within gut tissue microbiota	PD_whole_tree	C-Sum	C-Spr	0.92	none
Seasonal comparison within gut tissue microbiota	PD_whole_tree	C-Aut	C-Spr	0.22	none

Comparison	Index	Group 1	Group 2	Exact p-value	Adjustment
Seasonal comparison within gut tissue microbiota	PD_whole_tree	C-Aut	C-Sum	0.27	none
Seasonal comparison within gut tissue microbiota	PD_whole_tree	C-Win	C-Spr	0.84	none
Seasonal comparison within gut tissue microbiota	PD_whole_tree	C-Win	C-Sum	0.93	none
Seasonal comparison within gut tissue microbiota	PD_whole_tree	C-Win	C-Aut	0.3	none
Seasonal comparison within gut content microbiota	observed_species	N-Sum	N-Spr	0.144	none
Seasonal comparison within gut content microbiota	observed_species	N-Aut	N-Spr	0.211	none
Seasonal comparison within gut content microbiota	observed_species	N-Aut	N-Sum	0.561	none
Seasonal comparison within gut content microbiota	observed_species	N-Win	N-Spr	0.081	none
Seasonal comparison within gut content microbiota	observed_species	N-Win	N-Sum	0.776	none
Seasonal comparison within gut content microbiota	observed_species	N-Win	N-Aut	0.661	none
Seasonal comparison within gut content microbiota	chao1	N-Sum	N-Spr	0.14	none
Seasonal comparison within gut content microbiota	chao1	N-Aut	N-Spr	0.21	none
Seasonal comparison within gut content microbiota	chao1	N-Aut	N-Sum	0.57	none
Seasonal comparison within gut content microbiota	chao1	N-Win	N-Spr	0.08	none
Seasonal comparison within gut content microbiota	chao1	N-Win	N-Sum	0.78	none
Seasonal comparison within gut content microbiota	chao1	N-Win	N-Aut	0.66	none
Seasonal comparison within gut content microbiota	shannon	N-Sum	N-Spr	0.1691	none
Seasonal comparison within gut content microbiota	shannon	N-Aut	N-Spr	0.2395	none
Seasonal comparison within gut content microbiota	shannon	N-Aut	N-Sum	0.8092	none
Seasonal comparison within gut content microbiota	shannon	N-Win	N-Spr	0.6655	none
Seasonal comparison within gut content microbiota	shannon	N-Win	N-Sum	0.0032	none
Seasonal comparison within gut content microbiota	shannon	N-Win	N-Aut	0.1735	none
Seasonal comparison within gut content microbiota	simpson	N-Sum	N-Spr	0.276	none
Seasonal comparison within gut content microbiota	simpson	N-Aut	N-Spr	0.477	none
Seasonal comparison within gut content microbiota	simpson	N-Aut	N-Sum	0.69	none
Seasonal comparison within gut content microbiota	simpson	N-Win	N-Spr	0.444	none
Seasonal comparison within gut content microbiota	simpson	N-Win	N-Sum	0.032	none
Seasonal comparison within gut content microbiota	simpson	N-Win	N-Aut	0.151	none

Comparison	Index	Group 1	Group 2	Exact p-value	Adjustment
Seasonal comparison within gut content microbiota	PD_whole_tree	N-Sum	N-Spr	0.301	none
Seasonal comparison within gut content microbiota	PD_whole_tree	N-Aut	N-Spr	0.199	none
Seasonal comparison within gut content microbiota	PD_whole_tree	N-Aut	N-Sum	0.389	none
Seasonal comparison within gut content microbiota	PD_whole_tree	N-Win	N-Spr	0.021	none
Seasonal comparison within gut content microbiota	PD_whole_tree	N-Win	N-Sum	0.201	none
Seasonal comparison within gut content microbiota	PD_whole_tree	N-Win	N-Aut	0.909	none
Seasonal comparison within water microbiota	observed_species	H-Sum	H-Spr	0.2485	none
Seasonal comparison within water microbiota	observed_species	H-Aut	H-Spr	0.9214	none
Seasonal comparison within water microbiota	observed_species	H-Aut	H-Sum	0.0412	none
Seasonal comparison within water microbiota	observed_species	H-Win	H-Spr	0.086	none
Seasonal comparison within water microbiota	observed_species	H-Win	H-Sum	0.0037	none
Seasonal comparison within water microbiota	observed_species	H-Win	H-Aut	0.0107	none
Seasonal comparison within water microbiota	chao1	H-Sum	H-Spr	0.2422	none
Seasonal comparison within water microbiota	chao1	H-Aut	H-Spr	0.9049	none
Seasonal comparison within water microbiota	chao1	H-Aut	H-Sum	0.0404	none
Seasonal comparison within water microbiota	chao1	H-Win	H-Spr	0.0873	none
Seasonal comparison within water microbiota	chao1	H-Win	H-Sum	0.0037	none
Seasonal comparison within water microbiota	chao1	H-Win	H-Aut	0.0122	none
Seasonal comparison within water microbiota	shannon	H-Sum	H-Spr	0.27139	none
Seasonal comparison within water microbiota	shannon	H-Aut	H-Spr	0.02555	none
Seasonal comparison within water microbiota	shannon	H-Aut	H-Sum	0.0138	none
Seasonal comparison within water microbiota	shannon	H-Win	H-Spr	0.01094	none
Seasonal comparison within water microbiota	shannon	H-Win	H-Sum	2.6e-05	none
Seasonal comparison within water microbiota	shannon	H-Win	H-Aut	0.00018	none
Seasonal comparison within water microbiota	simpson	H-Sum	H-Spr	0.09636	none
Seasonal comparison within water microbiota	simpson	H-Aut	H-Spr	0.00029	none
Seasonal comparison within water microbiota	simpson	H-Aut	H-Sum	0.00166	none
Seasonal comparison within water microbiota	simpson	H-Win	H-Spr	0.00614	none

Comparison	Index	Group 1	Group 2	Exact p-value	Adjustment
Seasonal comparison within water microbiota	simpson	H-Win	H-Sum	7.4e-05	none
Seasonal comparison within water microbiota	simpson	H-Win	H-Aut	0.00116	none
Seasonal comparison within water microbiota	PD_whole_tree	H-Sum	H-Spr	0.1326	none
Seasonal comparison within water microbiota	PD_whole_tree	H-Aut	H-Spr	0.868	none
Seasonal comparison within water microbiota	PD_whole_tree	H-Aut	H-Sum	0.0151	none
Seasonal comparison within water microbiota	PD_whole_tree	H-Win	H-Spr	0.0883	none
Seasonal comparison within water microbiota	PD_whole_tree	H-Win	H-Sum	0.0037	none
Seasonal comparison within water microbiota	PD_whole_tree	H-Win	H-Aut	0.0069	none

Note: Pairwise p-values are reproduced from the vendor post hoc output files. The original files reported no multiple-testing adjustment (P_adjustment_method = none), so these pairwise values are presented for completeness and should be interpreted together with the overall Kruskal–Wallis tests in Supplementary Table S3a. Group codes denote niche–season combinations: H, water microbiota; N, gut content microbiota; C, gut tissue-associated microbiota. Spr, spring; Sum, summer; Aut, autumn; Win, winter.
