# Supplementary material for: Seasonal Dynamics of the Gut Microbiota of Ayu (Plecoglossus altivelis) Revealed by a Cross-Sectional Seasonal Survey in the Dajing Stream, Zhejiang Province, China
Source: Biology (Basel). 2026 Apr 11;15(8):605. doi: 10.3390/biology15080605 (PMC13114198; doi:10.3390/biology15080605)
Supplement: Supplementary file 1 [file biology-15-00605-s001.zip › SuppTable S1-S7/SuppTable_S4_PERMANOVA.pdf]

Supplementary Table S4. Full PERMANOVA results for seasonal and niche comparisons.

Note: PERMANOVA values were transcribed directly from the raw vendor adonis output files. Human-readable comparison labels are shown here for clarity.

Comparison	Distance metric	Df_between	Df_within	Pseudo-F	R^2	p-value	Source
Seasonal comparison within gut tissue microbiota	Bray_Curtis	3	8	1.3342	0.33349	0.118	Raw vendor adonis output
Seasonal comparison within gut tissue microbiota	Unweighted_UniFrac	3	8	1.0442	0.2814	0.208	Raw vendor adonis output
Seasonal comparison within gut tissue microbiota	Weighted_UniFrac	3	8	1.6112	0.37663	0.253	Raw vendor adonis output
Seasonal comparison within gut content microbiota	Bray_Curtis	3	8	1.3322	0.33314	0.021	Raw vendor adonis output
Seasonal comparison within gut content microbiota	Unweighted_UniFrac	3	8	1.129	0.29745	0.001	Raw vendor adonis output
Seasonal comparison within gut content microbiota	Weighted_UniFrac	3	8	1.9194	0.41853	0.048	Raw vendor adonis output
Seasonal comparison within water microbiota	Bray_Curtis	3	8	156.37	0.98323	0.001	Raw vendor adonis output
Seasonal comparison within water microbiota	Unweighted_UniFrac	3	8	3.938	0.59625	0.001	Raw vendor adonis output
Seasonal comparison within water microbiota	Weighted_UniFrac	3	8	1001.2	0.99734	0.001	Raw vendor adonis output
Spring comparison among gut tissue, gut content, and water microbiota	Bray_Curtis	2	6	4.6589	0.6083	0.003	Raw vendor adonis output
Spring comparison among gut tissue, gut content, and water microbiota	Unweighted_UniFrac	2	6	2.6357	0.46768	0.004	Raw vendor adonis output
Spring comparison among gut tissue, gut content, and water microbiota	Weighted_UniFrac	2	6	14.784	0.8313	0.01	Raw vendor adonis output
Summer comparison among gut tissue, gut content, and water microbiota	Bray_Curtis	2	6	4.4298	0.59622	0.023	Raw vendor adonis output
Summer comparison among gut tissue, gut content, and water microbiota	Unweighted_UniFrac	2	6	3.4999	0.53845	0.004	Raw vendor adonis output
Summer comparison among gut tissue, gut content, and water microbiota	Weighted_UniFrac	2	6	12.988	0.81236	0.008	Raw vendor adonis output
Autumn comparison among gut tissue, gut content, and water microbiota	Bray_Curtis	2	6	5.6373	0.65267	0.004	Raw vendor adonis output
Autumn comparison among gut tissue, gut content, and water microbiota	Unweighted_UniFrac	2	6	2.6349	0.4676	0.01	Raw vendor adonis output
Autumn comparison among gut tissue, gut content, and water microbiota	Weighted_UniFrac	2	6	26.577	0.89857	0.006	Raw vendor adonis output
Winter comparison among gut tissue, gut content, and water	Bray_Curtis	2	6	3.4488	0.5348	0.004	Raw vendor adonis output

Comparison	Distance metric	Df_between	Df_within	Pseudo-F	R^2	p-value	Source
microbiota							
Winter comparison among gut tissue, gut content, and water microbiota	Unweighted_UniFrac	2	6	2.1919	0.42218	0.005	Raw vendor adonis output
Winter comparison among gut tissue, gut content, and water microbiota	Weighted_UniFrac	2	6	24.51	0.89095	0.006	Raw vendor adonis output
Seasonal comparison of non-host stomach-content COI profiles	Bray_Curtis	3	8	2.8702	0.51838	0.01	Raw vendor adonis output
Seasonal comparison of non-host stomach-content COI profiles	Weighted_UniFrac	3	8	2.5503	0.48885	0.052	Raw vendor adonis output
Seasonal comparison of non-host stomach-content COI profiles	Unweighted_UniFrac	3	8	2.2378	0.45628	0.001	Raw vendor adonis output
