# Supplementary material for: Seasonal Dynamics of the Gut Microbiota of Ayu (Plecoglossus altivelis) Revealed by a Cross-Sectional Seasonal Survey in the Dajing Stream, Zhejiang Province, China
Source: Biology (Basel). 2026 Apr 11;15(8):605. doi: 10.3390/biology15080605 (PMC13114198; doi:10.3390/biology15080605)
Supplement: Supplementary file 1 [file biology-15-00605-s001.zip › SuppTable S1-S7/SuppTable_S5_PERMDISP.pdf]

Supplementary Table S5. PERMDISP results for seasonal and niche comparisons.

Comparison	Distance metric	Df_between	Df_within	F	p-value	Source note
Seasonal comparison within gut tissue microbiota	Bray_Curtis	3	8	8.5803	0.29204	Recalculated from original Bray-Curtis distance matrix (20,000 permutations; seed=0)
Seasonal comparison within gut content microbiota	Bray_Curtis	3	8	3.8588	0.38593	Recalculated from original Bray-Curtis distance matrix (20,000 permutations; seed=0)
Seasonal comparison within water microbiota	Bray_Curtis	3	8	26.0949	0.01005	Recalculated from original Bray-Curtis distance matrix (20,000 permutations; seed=0)
Spring comparison among gut tissue, gut content, and water microbiota	Bray_Curtis	2	6	23.9209	0.03214	Recalculated from original Bray-Curtis distance matrix (exact permutation over all unique 3+3+3 labelings)
Summer comparison among gut tissue, gut content, and water microbiota	Bray_Curtis	2	6	815.5333	0.00357	Recalculated from original Bray-Curtis distance matrix (exact permutation over all unique 3+3+3 labelings)
Autumn comparison among gut tissue, gut content, and water microbiota	Bray_Curtis	2	6	40.8516	0.02857	Recalculated from original Bray-Curtis distance matrix (exact permutation over all unique 3+3+3 labelings)
Winter comparison among gut tissue, gut content, and water microbiota	Bray_Curtis	2	6	460.7464	0.00357	Recalculated from original Bray-Curtis distance matrix (exact permutation over all unique 3+3+3 labelings)
Seasonal comparison of non-host stomach-content COI profiles	Bray_Curtis	3	8	4.0873	0.27909	Recalculated from original Bray-Curtis distance matrix (20,000 permutations; seed=0)

Note: Because the vendor result package did not include direct PERMDISP output files, these values were recalculated from the original Bray–Curtis distance matrices. Human-readable comparison labels are shown here for clarity
