# Supplementary material for: Seasonal Dynamics of the Gut Microbiota of Ayu (Plecoglossus altivelis) Revealed by a Cross-Sectional Seasonal Survey in the Dajing Stream, Zhejiang Province, China
Source: Biology (Basel). 2026 Apr 11;15(8):605. doi: 10.3390/biology15080605 (PMC13114198; doi:10.3390/biology15080605)
Supplement: Supplementary file 1 [file biology-15-00605-s001.zip › SuppTable S1-S7/SuppTable_S6_Shared_ASV.pdf]

Supplementary Table S6. Quantification of shared/core ASVs across seasons and niches.

Comparison	Metric	Value
Seasonal comparison within gut tissue microbiota	Shared_by_all_groups	184
Seasonal comparison within gut tissue microbiota	Unique_to_C-Spr	1508
Seasonal comparison within gut tissue microbiota	Unique_to_C-Sum	1755
Seasonal comparison within gut tissue microbiota	Unique_to_C-Aut	2506
Seasonal comparison within gut tissue microbiota	Unique_to_C-Win	1489
Seasonal comparison within gut content microbiota	Shared_by_all_groups	165
Seasonal comparison within gut content microbiota	Unique_to_N-Spr	2886
Seasonal comparison within gut content microbiota	Unique_to_N-Sum	2218
Seasonal comparison within gut content microbiota	Unique_to_N-Aut	1759
Seasonal comparison within gut content microbiota	Unique_to_N-Win	2208
Seasonal comparison within water microbiota	Shared_by_all_groups	64
Seasonal comparison within water microbiota	Unique_to_H-Spr	625
Seasonal comparison within water microbiota	Unique_to_H-Sum	285
Seasonal comparison within water microbiota	Unique_to_H-Aut	520
Seasonal comparison within water microbiota	Unique_to_H-Win	892
Spring comparison among gut tissue, gut content, and water microbiota	Shared_by_all_groups	74
Spring comparison among gut tissue, gut content, and water microbiota	Unique_to_C	1663
Spring comparison among gut tissue, gut content, and water microbiota	Unique_to_N	3075
Spring comparison among gut tissue, gut content, and water microbiota	Unique_to_H	752
Summer comparison among gut tissue, gut content, and water microbiota	Shared_by_all_groups	36
Summer comparison among gut tissue, gut content, and water microbiota	Unique_to_C	1880
Summer comparison among gut tissue, gut content, and water microbiota	Unique_to_N	2421
Summer comparison among gut tissue, gut content, and water microbiota	Unique_to_H	438
Autumn comparison among gut tissue, gut content, and water microbiota	Shared_by_all_groups	96
Autumn comparison among gut tissue, gut content, and water microbiota	Unique_to_C	2607

Comparison	Metric	Value
Autumn comparison among gut tissue, gut content, and water microbiota	Unique_to_N	1937
Autumn comparison among gut tissue, gut content, and water microbiota	Unique_to_H	708
Winter comparison among gut tissue, gut content, and water microbiota	Shared_by_all_groups	197
Winter comparison among gut tissue, gut content, and water microbiota	Unique_to_C	1581
Winter comparison among gut tissue, gut content, and water microbiota	Unique_to_N	2348
Winter comparison among gut tissue, gut content, and water microbiota	Unique_to_H	876

Note: Shared_by_all_groups denotes ASVs common to all groups within a comparison. Unique_to_* values denote ASVs unique to the indicated season or niche group. Group codes follow the nomenclature H/N/C with Spr/Sum/Aut/Win for season.
