# Supplementary material for: Seasonal Dynamics of the Gut Microbiota of Ayu (Plecoglossus altivelis) Revealed by a Cross-Sectional Seasonal Survey in the Dajing Stream, Zhejiang Province, China
Source: Biology (Basel). 2026 Apr 11;15(8):605. doi: 10.3390/biology15080605 (PMC13114198; doi:10.3390/biology15080605)
Supplement: Supplementary file 1 [file biology-15-00605-s001.zip › SuppTable S1-S7/SuppTable_S7_COI_reads_host.pdf]

Supplementary Table S7. Per-sample COI sequencing summary and host-read filtering statistics.

Note: Retained_feature_reads indicates the reads remaining after feature-table processing. Host_assigned_proportion_of_retained was calculated as Host_assigned_reads / Retained_feature_reads, and Non_host_reads indicates the reads retained after host-read removal.

Part 1. Per-sample COI sequencing summary.

Sample	Season	Clean sequences	Retained feature reads	Retained %	Bases (bp)	Avg. length (bp)
D-Spr1	Spring	126234	107783	85.38	39515453	313.03
D-Spr2	Spring	122317	109314	89.37	38276273	312.93
D-Spr3	Spring	129003	116033	89.95	40370890	312.95
D-Sum1	Summer	122100	106636	87.33	38186788	312.75
D-Sum2	Summer	103303	91348	88.43	32326494	312.93
D-Sum3	Summer	107317	95399	88.89	33593978	313.04
D-Aut1	Autumn	100257	93979	93.74	31384382	313.04
D-Aut2	Autumn	112131	91948	82.00	35022280	312.33
D-Aut3	Autumn	100054	89497	89.45	31295244	312.78
D-Win1	Winter	129758	107997	83.23	40791734	314.37
D-Win2	Winter	100175	95918	95.75	31349130	312.94
D-Win3	Winter	122244	93548	76.53	38460897	314.62

Part 2. Host-read removal summary.

Sample	Season	Host-assigned reads	Host-assigned % of retained	Non-host reads
D-Spr1	Spring	96038	89.10	11745
D-Spr2	Spring	105058	96.11	4256
D-Spr3	Spring	114126	98.36	1907
D-Sum1	Summer	96538	90.53	10098
D-Sum2	Summer	81806	89.55	9542
D-Sum3	Summer	93250	97.75	2149

Sample	Season	Host-assigned reads	Host-assigned % of retained	Non-host reads
D-Aut1	Autumn	92273	98.18	1706
D-Aut2	Autumn	26645	28.98	65303
D-Aut3	Autumn	56466	63.09	33031
D-Win1	Winter	38841	35.96	69156
D-Win2	Winter	74744	77.92	21174
D-Win3	Winter	31377	33.54	62171
