# Supplementary figures and images for: Seasonal Dynamics of the Gut Microbiota of Ayu (Plecoglossus altivelis) Revealed by a Cross-Sectional Seasonal Survey in the Dajing Stream, Zhejiang Province, China
Source: Biology (Basel). 2026 Apr 11;15(8):605. doi: 10.3390/biology15080605 (PMC13114198; doi:10.3390/biology15080605)

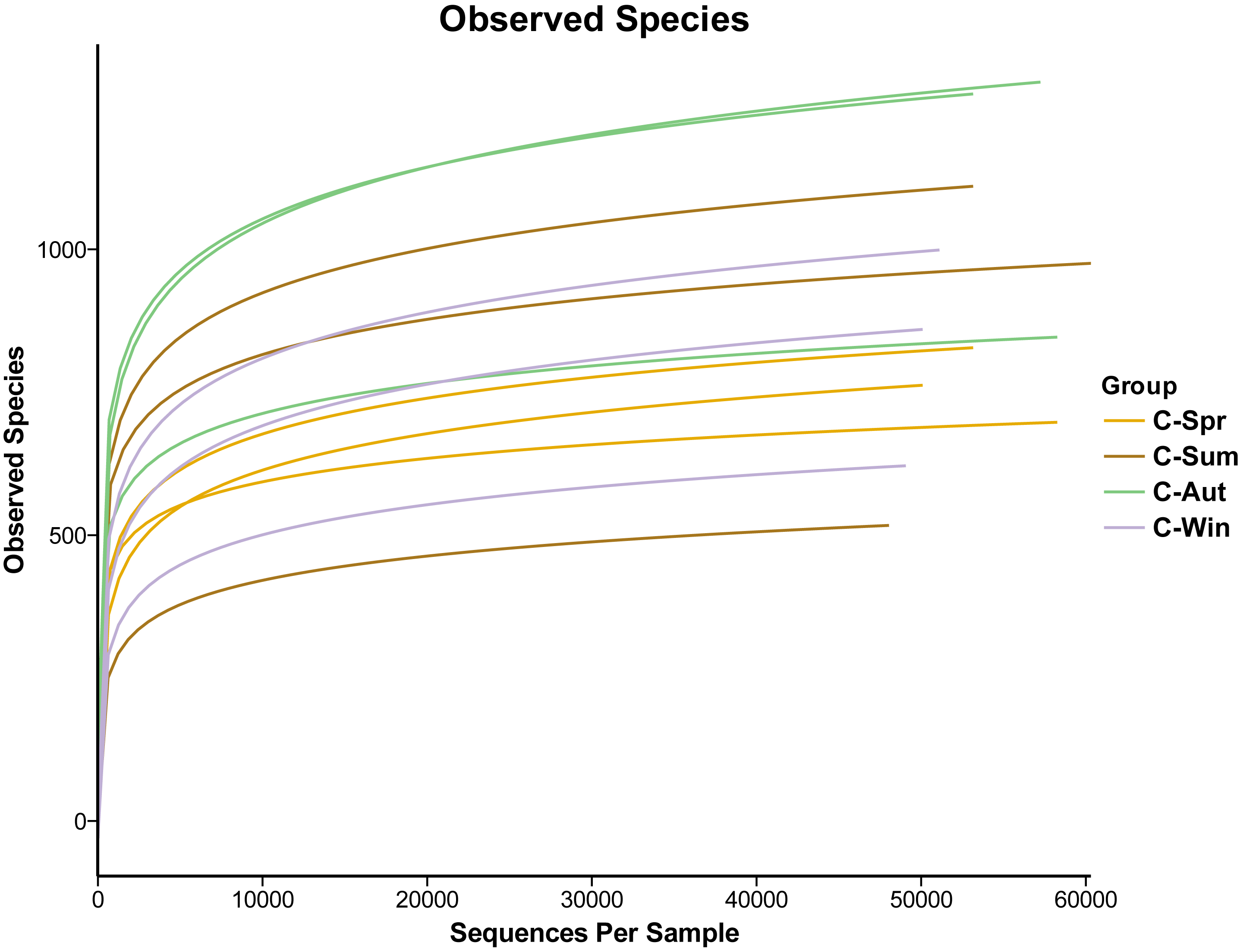

Supplement: Supplementary file 1 [file biology-15-00605-s001.zip › Supplementary_FigureS1-S7/Supplementary Figure S1_C_season_ rarefaction curve.tif]

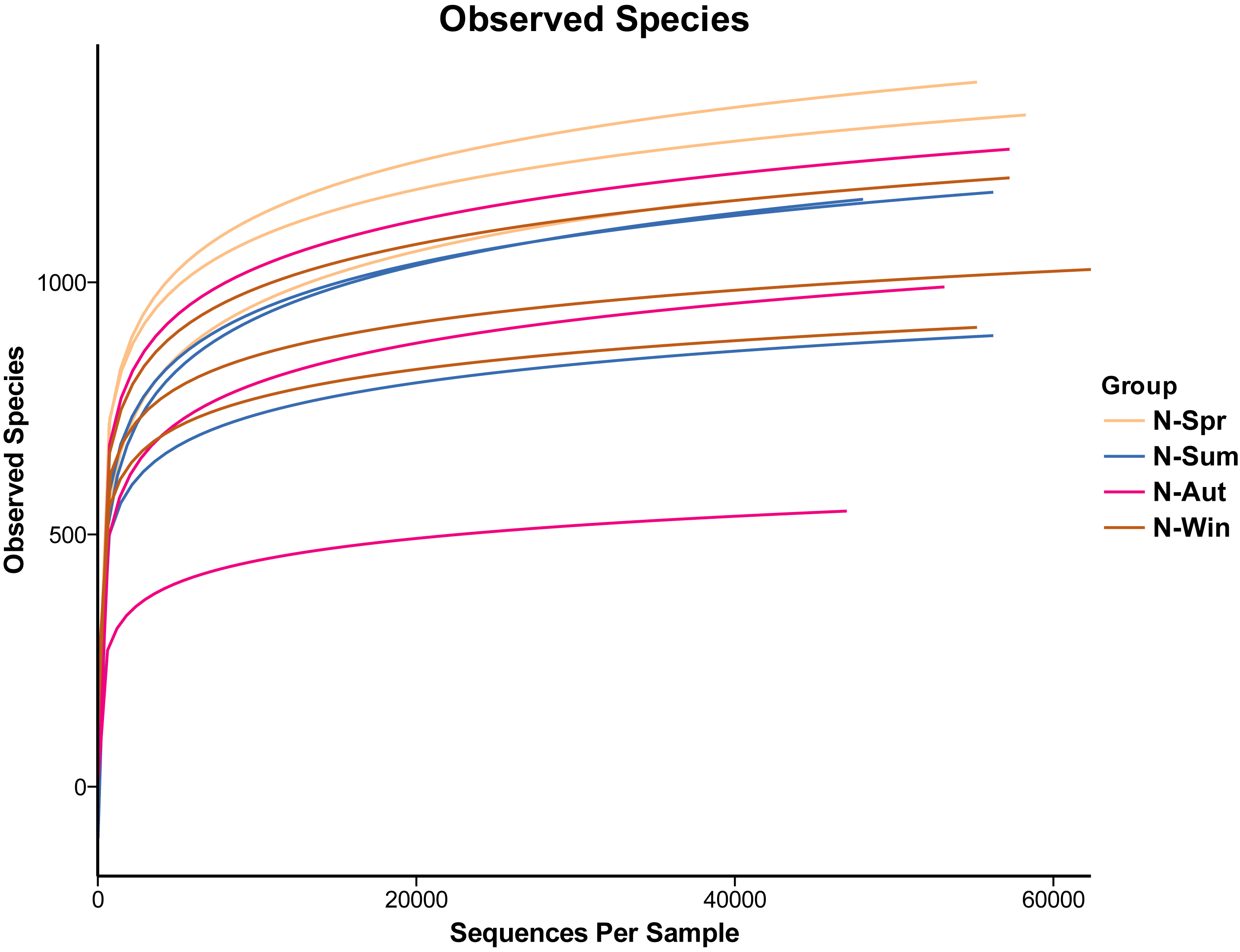

Supplement: Supplementary file 1 [file biology-15-00605-s001.zip › Supplementary_FigureS1-S7/Supplementary Figure S2_N _season_rarefaction curve.tif]

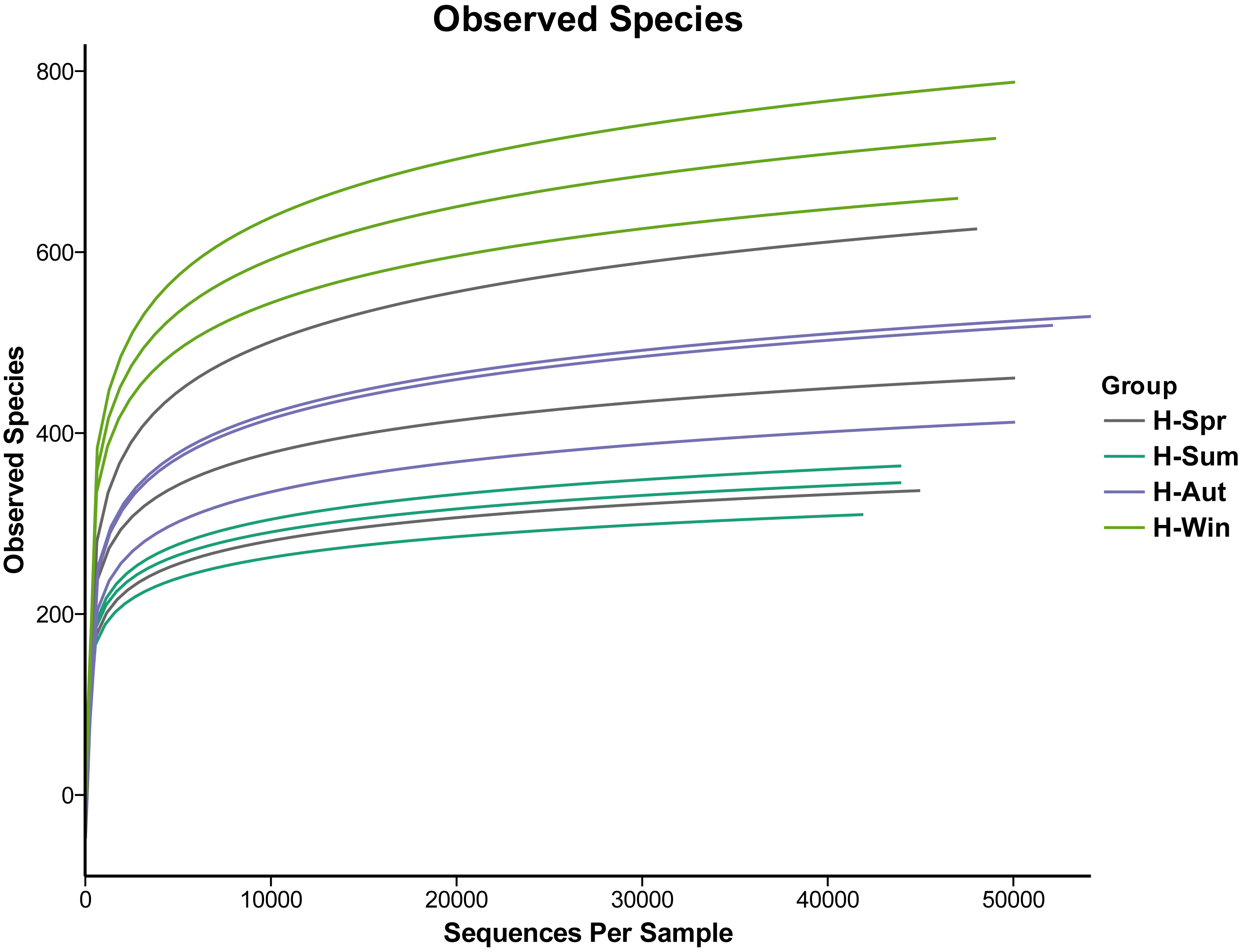

Supplement: Supplementary file 1 [file biology-15-00605-s001.zip › Supplementary_FigureS1-S7/Supplementary Figure S3_H _season_rarefaction curve.tif]

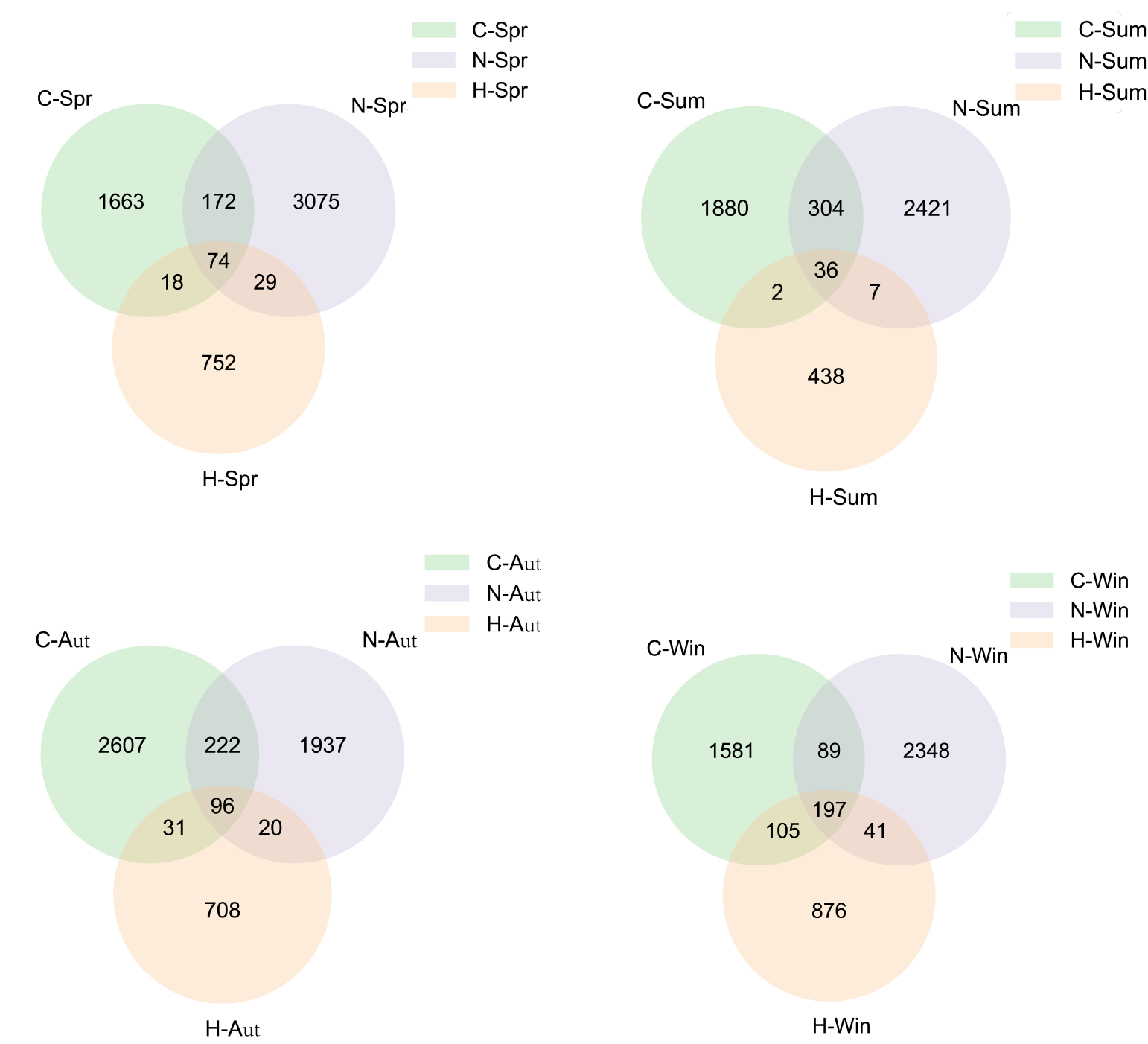

Supplement: Supplementary file 1 [file biology-15-00605-s001.zip › Supplementary_FigureS1-S7/Supplementary_Figure_S7_HNC_shared_unique_ASVs_season.tif]
